# Continental-Scale Assessment of Risk to the Australian Odonata from Climate Change

**DOI:** 10.1371/journal.pone.0088958

**Published:** 2014-02-13

**Authors:** Alex A. Bush, David A. Nipperess, Daisy E. Duursma, Gunther Theischinger, Eren Turak, Lesley Hughes

**Affiliations:** 1 Dept. of Biological Sciences, Macquarie University, Sydney, New South Wales, Australia; 2 Office of Environment and Heritage, Sydney, New South Wales, Australia; Ecole Normale Supérieure de Lyon, France

## Abstract

Climate change is expected to have substantial impacts on the composition of freshwater communities, and many species are threatened by the loss of climatically suitable habitat. In this study we identify Australian Odonata (dragonflies and damselflies) vulnerable to the effects of climate change on the basis of exposure, sensitivity and pressure to disperse in the future. We used an ensemble of species distribution models to predict the distribution of 270 (85%) species of Australian Odonata, continent-wide at the subcatchment scale, and for both current and future climates using two emissions scenarios each for 2055 and 2085. Exposure was scored according to the departure of temperature, precipitation and hydrology from current conditions. Sensitivity accounted for change in the area and suitability of projected climatic habitat, and pressure to disperse combined measurements of average habitat shifts and the loss experienced with lower dispersal rates. Streams and rivers important to future conservation efforts were identified based on the sensitivity-weighted sum of habitat suitability for the most vulnerable species. The overall extent of suitable habitat declined for 56–69% of the species modelled by 2085 depending on emissions scenario. The proportion of species at risk across all components (exposure, sensitivity, pressure to disperse) varied between 7 and 17% from 2055 to 2085 and a further 3–17% of species were also projected to be at high risk due to declines that did not require range shifts. If dispersal to Tasmania was limited, many south-eastern species are at significantly increased risk. Conservation efforts will need to focus on creating and preserving freshwater refugia as part of a broader conservation strategy that improves connectivity and promotes adaptive range shifts. The significant predicted shifts in suitable habitat could potentially exceed the dispersal capacity of Odonata and highlights the challenge faced by other freshwater species.

## Introduction

Climate change is a major challenge for biodiversity within all ecosystems e.g. [1]. River and stream ecosystems appear to be particularly sensitive [2], [3] and face numerous challenges including the direct impacts of warming temperatures [4], [5], altered hydrology [6], the increased frequency of floods and drought [7], sea-level rise [8] and multiple other anthropogenic stressors [9]–[11]. Climate change is projected to have impacts across all scales of organisation in freshwater ecosystems, from effects on genetic diversity [12] to community composition [13]. Observations of climate change impacts are increasing rapidly, including shifts in phenology [14], shifts in distribution [15], [16] and shifts in community composition and structure e.g. [17], [18].

Understanding how best to conserve biodiversity under climate change is a major challenge, in part due to a poor understanding of species distribution i.e. the Wallacean shortfall [19]. Freshwater diversity in particular has often been overlooked within the wider terrestrial landscape [20] and conservation focus is biased to vertebrates, despite invertebrates contributing the bulk of biodiversity [19]. To overcome the shortfall in data, Species Distribution Models (SDMs) have become popular tools because they can maximise the use of the limited records we have to predict the suitability of habitat in the wider landscape e.g. [21]. By extending projections through time they can be used to predict the threat posed by climate change [22]–[24]. If the area of suitable habitat is predicted to be dramatically reduced by climate change then that species may face significant risk of extinction in the future as conditions become increasingly marginal. The resolution and complexity of geographic data for river systems is continuously improving and as a result the number of studies applying SDMs to freshwater taxa has increased rapidly in recent years, with applications to fish [25], [26], platypus [27], and aquatic invertebrates [28], [29].

In this study we described the distribution of Australian Odonata (dragonflies and damselflies. Air temperature has increased 0.9°C in Australia since 1910, with most warming occurring since 1970, and includes more temperature extremes that match model expectations [30], [31]. Predicted changes to rainfall and hydrology will mean some regions experience significant deficits and others increased variability in coming decades [32]. Previous studies have shown that Odonata appear to be suited to assessing the impacts of climate change because their development is strongly temperature dependent [4], their distribution is not dependent on other species [33], and they are sensitive to climatic factors e.g. [34], [35], a key assumption when using SDMs for climate change assessments. Odonata have been successfully modelled for conservation purposes [36], and within studies of climate change effects on macroinvertebrates [16]. There have also been many reported changes in odonate ranges consistent with a response to recent climate change e.g. [37]. In addition, Odonata were selected because, among the major orders of aquatic invertebrates, they occur in all Australian surface waters, their taxonomy is relatively well known, and comprehensive occurrence data are available [38].

We assessed threats to species based on a combination of their likely exposure to climatic change, their sensitivity to climatic factors, and the relative importance of dispersal capability [39], [40]. This study assesses the threat to Odonata, an invertebrate group widespread across the Australian continent and models changes in suitability at a spatial scale appropriate for conservation management of freshwater systems. In the absence of measured trait-data to characterise species’ adaptive capacity, typical for invertebrate taxa (but see [41]), we used the distance habitats are predicted to shift in the future to describe the pressure on species to disperse and track suitable conditions. This approach does not therefore describe species’ adaptive capacity *per se*, but identifies the species that would face significantly greater risk if they were *not able* to disperse as fast as their suitable habitats shift. Of the species included in the models, we identified those Australian Odonata most vulnerable to climate change across multiple criteria, and identified the specific locations most important for conservation of the most vulnerable species.

## Materials and Methods

### Species Data

Records of odonate distributions were collated from a diverse range of sources including all state and museum collections, government survey records, local catchment authorities, scientific literature and private collectors. For several collections these records were entered into digital format for the first time, significantly increasing the overall number of records available (Table S1). Locality records and taxonomic identification were verified for accuracy as much as possible using habitat descriptions within metadata and expert advice of collectors and museums [42], [43]. Although outlying records can influence model fitting, where doubt existed over observation validity the records were removed. Decisions on record validity incorporated factors such as date recorded, life stage (favouring larvae over adults) and gender (females over males). For example, some species had adult males recorded far beyond their usual range (300 km+) in highly arid environments, presumably following an unusually heavy period of rainfall. Populations in these areas are unlikely to be self-sustaining for even a few generations and the records were removed from the dataset.

The completed dataset included over 32,000 occurrence records from approximately 12,100 localities. Of the 324 Australian Odonata, modelling included 197 species recorded from 30 or more subcatchments, and a further 76 species that were treated as “Uncommon” (15–30 subcatchments) [44]. The majority of records were collected within the last 20 years (95%), but records as far back as 1950 were also used in the case of some uncommon species where native vegetation was still intact, and they had not been recorded in more than 14 subcatchments more recently. A number of species distribution modelling studies have used low numbers of records to successfully predict distributions e.g. [45], [46], and by adjusting parameters so models were not over-fitted we were able to include uncommon species e.g. [16], [47]. Nonetheless, approximately 51 species were recorded from fewer than 15 subcatchments and were not included in this study.

### Environmental Data

Climate change projections were based on Representative Concentration Pathways (RCPs), being the standardised warming trajectories due to be used in the Intergovernmental Panel on Climate Change’s Fifth Assessment Report in 2013 [48], [49]. The RCPs used in this study describe a range of stabilisation, mitigation and non-mitigation pathways that under medium or high emissions scenarios result in radiative forcing reaching 6 and 8.5 W/m^2^ respectively by 2100, equivalent to global average temperatures increasing 3.0 and 4.9°C [50]. Coarse resolution climate data were provided by the Tyndall Centre, University of East Anglia, UK (available at http://climascope.wwfus.org). Based on the study by Fordham et al. [51] we selected an ensemble of the seven global climate models (GCMs) most successful at reproducing the recent global and regional precipitation patterns of Australia (specifically CCSR-MIROC32MED, CSIRO-MK30, GFDL-CM20, MPI-ECHAM5, MRI-CGCM232A, UKMO-HADCM3 and UKMO-HADGEM1). The data were 10-year averages centered around 2055, and 2085, for RCP6 (medium emissions scenario) and RCP8.5 (high emissions scenario). Lower emissions scenarios were omitted in this study as all indications suggest achieving the necessary reductions are unlikely [52]. Research has shown that climate ensembles perform better than any single GCM in simulating observed conditions [53], and multiple scenarios are useful to span the range of uncertainty in predicting future climates [54]. Monthly RCP data were statistically downscaled to a 1 km^2^ resolution, independent of elevation, using a cubic spline of the anomalies (deviance from modelled current and modelled future) and these anomalies were applied to a current climate baseline of 1950 to 2000. The current climate data were sourced from Worldclim (www.worldclim.org) and the data were created as defined in Hijmans et al. [55]. The same method was used to create bioclimatic variables from the downscaled future climate data. All downscaling and bioclimatic variable generation was performed using the ‘climates’ package [56] in R v.2.15 [57].

Rather than using gridded data, models were based on the stream network from the National Catchment and Stream Environment Database V.1.1.3, part of the Australian Hydrological Geospatial Fabric [58]. When predicting habitat suitability in river networks, organising the modelling environment and predictor variables to reflect the structure of a freshwater system is important because it can influence the accuracy of freshwater SDMs without necessarily affecting performance metrics [59]. Catchment boundaries were coded hierarchically using the Pfafstetter classification system that defines 1.4 million stream subcatchments at the continental scale. Climate data were aggregated to the same stream subcatchments. Mean annual runoff was generated by James et al. [45] for the same stream network and same future climate scenarios using a bucket model outlined by Donohue et al. [60]. Local differences in precipitation can be poor proxies for changes to runoff [61], and hydrological forecasts can therefore greatly improve projections of habitat suitability for freshwater species.

We used ENMTools [62] and Maxent [63] to calculate model AIC (Akaike Information Criterion [64]) and to rank variables for approximately 20% of the species. We did not observe a significant difference in variable selection among major taxonomic families or between species that could be associated with still or flowing waters, but variable selection did differ among species assigned to different geographic regions (see File S1). By selecting models with the lowest AIC the array of climatic, hydrological and topographic variables was reduced to eight. The predictor variables used included three temperature variables (annual mean, seasonality, and minimum of the coldest month), three precipitation variables (precipitation of the wettest and driest quarters, and seasonality), one hydrological (mean accumulated flow) and one topographical (valley confinement). Valley confinement is a useful proxy for the sedimentation characteristics of a subcatchment and particularly useful for upland catchments [65]. Most species were best modelled using seven variables, although uncommon species in each region were modelled using five. Selection only varied geographically among groups based on the use of precipitation in either dry or wet quarters. Australia has very few Odonata exclusively associated with standing water and this may be why presence of standing water bodies such as lakes did not rank highly [38]. In the case of two dune lake specialists, the density of lakes and extent of sandy soils were included in models, although this did not significantly improve model scores.

### Habitat Suitability Modelling

Odonata distributions were modelled using an ensemble of five commonly used algorithms within the package *BIOMOD* 2 in R [66]. Algorithms included generalised linear models (GLM), generalised boosted models (GBM), generalised additive models (GAM), multivariate adaptive regression splines (MARS), and Maxent [67]. All models were run with 10 replicates, using a standard 70/30 split for training and testing data. Algorithms were run using their default settings and adjusted as follows: GLM, polynomial terms were ranked by AIC; GBM, fourfold cross-validation and a maximum of 2,000 trees; GAM, a spline function with a degree of smoothing of four and 10,000 pseudo-absences.

Model evaluation was conducted using the standard measure of area under the receiver operating characteristic curve (AUC), and the True Skills Statistic (TSS). The sole use of AUC in SDM studies has received some criticism, particularly when models are fitted across large areas [68], [69], and so TSS was used in weighting model importance for ensemble projection [66], and maximised when selecting a suitable threshold to perform binary transformations [70], [71]. AUC scores range from 0 to 1; values of 0.5 indicate a performance no better than random, whereas 1 reflects perfect model accuracy. TSS scores range from −1 to 1, with 0 indicating no skill and 1 a perfect ability to distinguish positive and false scores.

The majority of Odonata records in Australia were distributed through the more mesic coastal regions, and there was a bias in their proximity to urban settlements, some major rivers and highways. As the ranges of most species are regionally restricted, the use of pseudo-absences from the entire study area would have led to exaggerated discrimination statistics [68], [69], and less informative models [72]. Pseudo-absences were selected from background points where other species had been collected within a 300 km radius of a species’ presence record [73], the maximum range we considered available to dispersing Odonata under current conditions. Pseudo-absences were supplemented where necessary by random selection to standardise the total across species. By reducing the overall extent of pseudo-absences, the model projections are more likely to extrapolate beyond the known species-environment relationships, potentially overestimating suitability in distant locations [74]. To counter extrapolation we constrained projections using environmental clamping that reduced the suitability of a subcatchment when more than one environmental factor was outside the limits used in model construction [63]. The clamping allowed some reasonable extrapolation of distributions to fill gaps in current habitat, but constrained suitability under future projections to reflect similar environmental conditions to the present.

Model performance based on TSS (0.827+/−0.124) and AUC was typically high (0.946+/−0.06), although TSS scores were more variable (see File S1 and Table S2). TSS scores were lowest among several common continental species, but they were retained after closer examination showed that their poor scores were the result of misclassification only in the arid zone where the patchy nature of waterholes made assessment difficult. However, three species with highly restricted current distributions were subsequently removed from the analysis because of high variation in projections from different models, particularly for future climate change scenarios. For the 270 remaining species modelled, the treatment of species as uncommon did not significantly influence the predicted overall change in habitat extent (t_(89)_ = −0.09, p = 0.926), but there was an increase in model performance commonly observed for narrow range species [68]. All projections of individual species presented in this study are freely available on request from the corresponding author.

### Vulnerability Assessment

**Figure 1:**
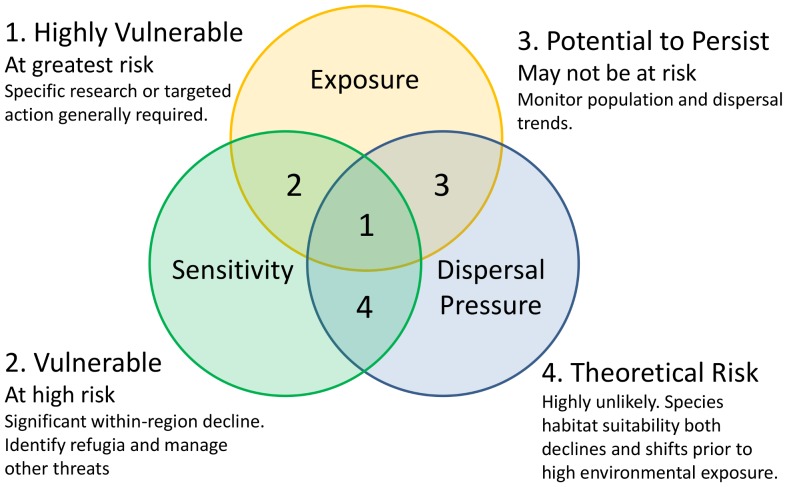
Categories of vulnerability to climate change. The effects of climate change on a species were based on three components: exposure, sensitivity and dispersal pressure. Possible adaptation options are given for species at risk under multiple components (adapted from [40]).

We determined species vulnerability to climate change based on three components; exposure (the extent to which a species’ currently occupied physical environment will change), sensitivity (the extent to which suitable habitat is lost) and dispersal pressure (the reliance on dispersal to avoid further negative impacts); Fig. 1 and [40]. Species at risk across all components were classified as highly vulnerable (Category 1). Species that are not required to disperse long distances but are still exposed and sensitive to change are considered vulnerable (Category 2). If a species is exposed to climate change and alternative suitable habitats are available but require significant dispersal, it was classified as having the potential to persist (Category 3). It is also possible, though unlikely in a modelled environment, for a species to experience a significant decline and distributional shift before becoming significantly exposed to environmental change (Category 4). A detailed example of the assessment process is available in File S2.

For each climate scenario and time period, exposure was calculated as the average number of standard deviations (SD) that conditions are projected to shift in the future across a species’ current modelled habitat. A change of one to two SDs in exposure meant 67–97.5% of a species habitat would be outside the current environmental extent. We assumed that species have evolved to cope with the inter-annual variation within their current environment. A change of two SDs was therefore considered a reasonable limit, beyond which the likelihood that a species would adapt *in situ* was very low [75], [76]. The mean and seasonality of annual temperature and precipitation, mean annual flow, and sea level rise were used as measures of exposure. A species was considered vulnerable if its exposure was above two SDs for any climate or hydrological factor, or if it was exposed above one SD for multiple factors. Exposure of a species’ suitable habitat to sea-level rise was also considered important if 10% of the habitat was within 1 m of sea level [77].

Species’ sensitivity was calculated using the methods described in Crossman et al. [78] as the ratio between the change in habitat suitability, and the future scenario total suitability. Change in a species’ distribution was based on the sum of habitat suitability over all streams in the future, subtracted from the sum of suitability for streams under current climate. Suitability scores below the species TSS-threshold were not included. Species with negative sensitivity values are likely to expand their range or have higher overall suitability in the future, whereas higher values occur when the species’ habitat either contracts in area, or becomes less suitable. Species with sensitivity ratios above one were considered highly vulnerable.

### Dispersal

In addition to exposure and sensitivity, the adaptive capacity of a species can also affect vulnerability. Dispersal is a key aspect of adaptive capacity because it affects the proportion of environmentally suitable habitat that a species can occupy, both now and in the future. Dispersal constraints were initially used to prevent highly unlikely scenarios requiring long-distance movements and improve upon standard no- and full-dispersal comparisons [79]. As the raw species model was constrained using a relatively high dispersal rate the estimate of vulnerability was considered conservative. The analysis of the impact of dispersal capacity on species vulnerability was therefore made in relation to this upper rate. Species were considered more vulnerable if suitable habitat in the future was distant from current records, or if the extent of suitable habitat rapidly declined when the dispersal threshold was reduced (see examples in File S2).

Measuring dispersal capacity directly is extremely difficult but studies of genetic population structure in aquatic insects suggest regular movement does occur across catchment divisions [80]. Migratory dragonflies can move 12 km per day [81] and as the climate has changed over the past few decades, there have been an increasing number of reports of species dispersing considerable distances to colonise new regions; e.g. *Anax imperator* (Leach, 1815) 88 km per year [82]. Nonetheless, most species are likely to disperse much shorter distances. Six European species studied by Jaeschke et al. [83] disperse between 0.5 and 14 km per year and the 37 non-migratory British species studied by Hickling et al. [84] expanded north by an average 6.8 km per year.

**Figure 2:**
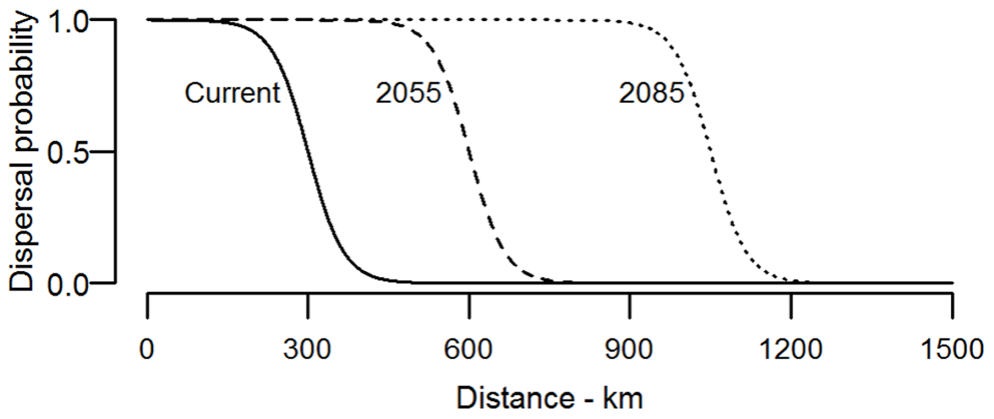
Modelled probability of species dispersal with distance from known records. Under current conditions (solid line) suitability is reduced around 300 km, and extended to 630 km (2055) and 1080 km (2085) under future climate change scenarios.

We restricted the area of suitable habitat available to a species based on a cost-weighted distance, and a dispersal kernel. The cost-weighted distance calculates a least-cost path across a 1 km grid that determines the cost of movement (done in ArcMap 10.1). Distance from recorded observations of a species to the centroid of other streams could be modified by altering the cost of movement across surfaces such as open water [85]. We then used a dispersal kernel based on a four-parameter logistic curve to model declining dispersal probability in an ecologically relevant way (Fig. 2). The dispersal kernel converted the cost-weighted distances to a value between 0 and 1 that indicates the probability of dispersal to that stream from known presences [78], [86]. The threshold distance and decay rate of the dispersal curve were varied so that weighting suitability scores by dispersal probability are reduced at distant locations beyond the threshold.

The choice of appropriate threshold and dispersal cost was based on the initial observation that many Odonata in Victoria have not been recorded in Tasmania, about 200 km away across the Bass Strait. However, a 200 km threshold would have prevented continuous distribution of some species in northern and central Australia where gaps are most likely a reflection of low sampling. As a result, we doubled the cost of crossing open water, but increased the threshold to 300 km, thereby allowing continuous mainland distributions and still constraining species occurrence in Tasmania if they currently only occur on the mainland. Under future projections, potential range shifts were allowed to occur by increasing the threshold distances to 630 and 1080 km for 2055 and 2085 respectively. These distances were equivalent to an expansion of 15 km year^−1^ from their current recorded position; a rate observed in the damselfly *Sympecma fusca*, that has responded rapidly to climate change in Sweden [82]. Interestingly the observed rate of expansion in the study by Hickling et al. [84] was not related to body size, indicating Anisoptera may not disperse more rapidly than Zygoptera [87]. In the absence of data for Australian species, this seemed an appropriate upper limit for this analysis [83].

To assess vulnerability based on the predicted pressure on a species to disperse, we split the assessment into two parts: the mean distance of habitat shifts, and the dependence of the sensitivity weighting on dispersal thresholds. First, we compared the mean distance from recorded observations of each species to all suitable habitat in their current and future modelled ranges using a Wilcoxon rank sum test. Species scored 0 if suitable habitat was not significantly further away from observed records in the future than the present, 1 if the difference was significant (*p* = 0.05 ∼2 SDs), and 2 if the difference was over three SDs, indicating decreasing overlap of habitats, or potentially greater fragmentation. The second approach identified the importance of rapid dispersal for a species by estimating at what point a reduction in dispersal ability from the conservative estimate of 15 km year^−1^ would significantly reduce the habitat available based on the sensitivity weight. The dispersal thresholds were split into 30 levels with 10 high, medium and low thresholds between the current habitat limit (300 km) and future threshold (630 or 1050 km depending on the scenario) (see examples in File S2). Sensitivity weights increased as suitable habitat was successively removed and we estimated the rate of change from the slope of a regression between threshold distance (log transformed) and the sensitivity weight. A species was given a score of 3, 2 or 1 if the slope was less than one for high, medium or low thresholds respectively, and zero if it was not. Thus species whose future suitable habitats are concentrated in distant regions are considered more vulnerable because small reductions (0.5–5 km year^−1^) in dispersal capacity would significantly reduce the availability of suitable habitat. Thus combined with the habitat shift score, a maximum of 5 points was available, and species that scored three or more were considered vulnerable.

Finally, separate to the two measurements of dispersal ability above, we also considered the possibility that the Bass Strait could remain a barrier to species shifting their distributions to Tasmania under climate change. This time the dispersal kernel was kept constant, but by increasing the cost of movement across the sea to 100 times that of land the dispersal kernel then acts to remove all potentially suitable habitat from Tasmania for species not already recorded there. Although some Odonata occur either side of the Bass Strait (e.g. the damselfly *Hemiphelbia mirabilis*), most do not, and we compared the sensitivity and vulnerability scores of species affected by this change.

### Conservation Priorities

The importance of all subcatchments to the conservation of vulnerable species was calculated for both highly vulnerable (Category 1) and vulnerable (Category 2) species for each time and emissions scenario. The score for all streams was the sum of habitat suitability weighted by the sensitivity weighting for that species in each scenario [78]. Thus, subcatchments scored highly if they contained suitable habitat for many vulnerable species, or for species that had experienced major declines in habitat suitability elsewhere.

## Results

**Figure 3:**
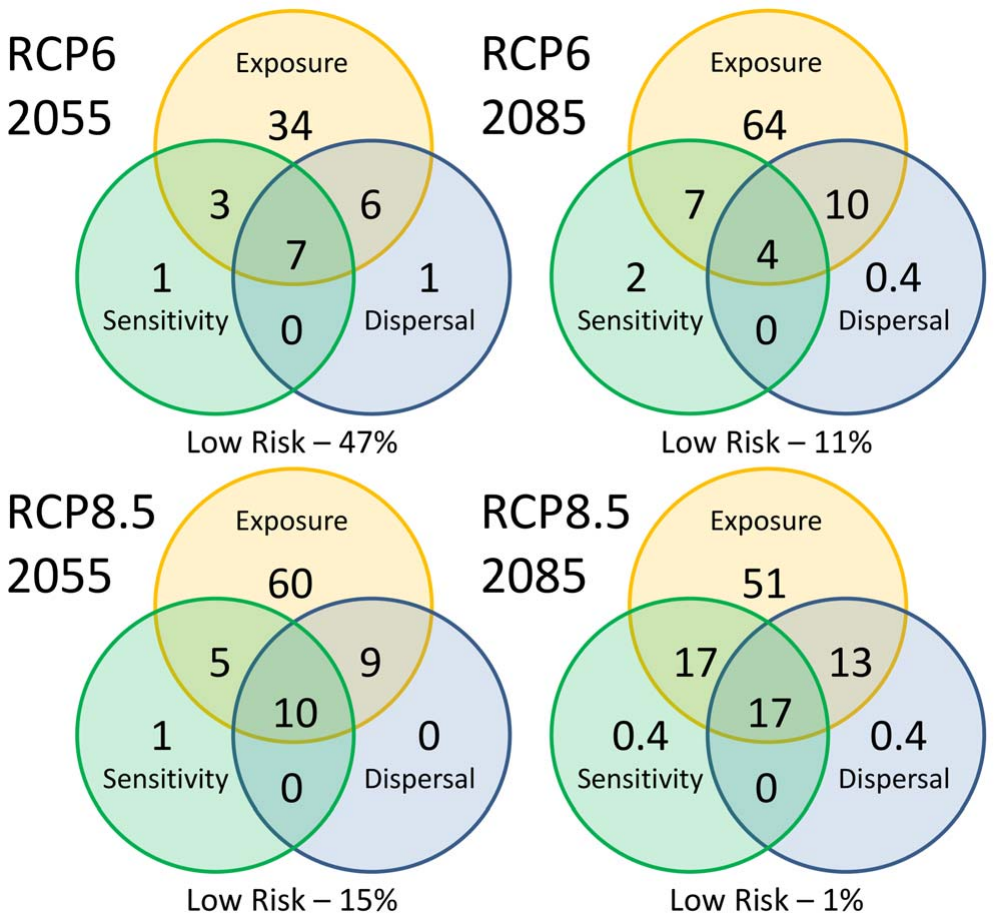
Percentage of species (*n* = 270) found to be vulnerable to climate change according to their exposure, sensitivity and predicted pressure to disperse. Species are most vulnerable if they are at risk in all components (Category 1).

**Figure 4:**
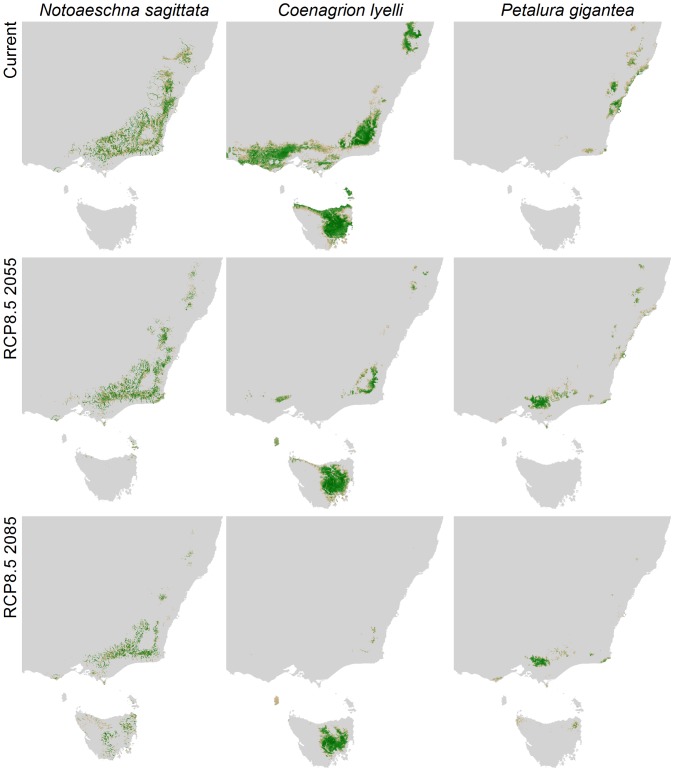
Predicted suitable habitat in south-eastern Australia under current climate and 2055 and 2085 using emissions scenario RCP8.5 for *Notoaeschna sagittata*, *Coenagrion lyelli* and *Petalura gigantea*. High suitability is in dark green.

Between 56 and 69% of species are predicted to experience an overall decline in habitat extent by 2085 depending on emissions scenario. Using the RCP 8.5 scenario, 17% of species were classified as vulnerable by 2085 (Category 2) due to high exposure to climatic change and significant declines in habitat suitability. A further 17% were classified as highly vulnerable (Category1) because to occupy suitable habitats they also need to disperse long distances (Fig. 3 and Fig. 4).

### Exposure

Environmental conditions shifted beyond the range experienced by 50–95% of species in their current suitable range under future climate change. By 2085 the current distribution of 30–61% of species was two SDs outside their current mean annual temperature range and two SDs outside the current range of annual flow in 59–71% of species. In all, 39–65% of species were exposed over multiple factors, and the number of factors to which a species was exposed was higher among uncommon species (t_(93)_ = −7.62, p =  <0.001). The species with the greatest exposure to potential change was *Archiargiolestes parvulus* (Watson, 1977), exposed across four factors as well as sea level rise. A 1 m rise in sea level was influential (loss>10%) for 44 species that on average lost 17% (SD 7.8, max 37%) of their suitable habitat due to this factor alone.

### Sensitivity

Species whose habitat was predicted to either contract substantially or to become significantly less suitable had a higher sensitivity weight. The predicted range of sensitivity scores reflects a broad range of potential responses from considerable expansion (e.g. *S* = −0.84, +500% for *Camacinia othello* Tillyard 1908) to near extinction (e.g. S = 30.5, −97% for *Lathrocordulia metallica* Tillyard 1911). Under both RCP6 and RCP8.5 emissions scenarios, six species (*Austroaeschna ingrid* Theischinger 2008, *Austroaeschna muelleri* Theischinger 1982, *Hemigomphus cooloola* Watson 1991, *Indolestes obiri* Watson 1979, *Lestoidea lewisiana* Theischinger 1996, *Nososticta pilbara* Watson 1969) are predicted to lose all suitable habitat by 2055. Fifteen species (including the six above) are predicted to have no suitable habitat remaining by 2085. Sensitivity weight was not correlated with overall habitat extent (r^2^ = 0.313) because there could be significant losses or gains in suitability as well. It was also not highly correlated with loss of current suitable habitat (r^2^<0.2) because many species were assumed to be able to colonise new suitable habitats.

### Dispersal Pressure

When relatively rapid movement (15 km year^−1^) using the dispersal kernel was assumed, most species were projected to be able to shift to higher latitudes (68% >1° by 2085–RCP8.5) or altitudes (46 Wet Tropics species move 245 m higher on average by 2085–RCP8.5), consistent with the exposure to rising temperatures. For example under the RCP8.5 scenario, 85 species were projected to potentially shift their distributions an average of 370 km by 2085 (max. species average  = 862 km). Successful transitions to these new habitats are less likely with increasing distance and we scored species vulnerability based on both distance travelled, and the impact of distance threshold on the species overall sensitivity. Of the 85 species above, 31 were projected to experience significant declines if the dispersal rate was reduced by just 0.5 to 5 km year^−1^. Manipulation of the dispersal kernel also showed that some species could be more vulnerable in the mid-term (2055) than under long-term projections (2085) because they needed to disperse long distances by 2055 to reach suitable habitat. This is partly the reason why the proportion of species in Category 1 is higher under scenario RCP6 in 2055 than 2085.

This assessment chose to rank each of the three components of vulnerability equally, and therefore only species at risk in all components were classified as highly vulnerable (i.e. Category 1 Fig. 4a). However, Category 2 species from the far south-west of the continent and from Tasmania do not have the option of shifting to habitats further south, and likewise suitable habitat conditions for species in the Wet Tropics are not predicted to become available elsewhere, meaning the species are inherently dispersal limited by the landscape [40]. Despite being highly exposed and sensitive to change, the lack of opportunity for movement meant habitats declined *in situ*, and dispersal capacity may be unlikely to contribute to greater vulnerability (Fig. 4b). In some cases, the overall decline (and sensitivity score) in suitable habitat was greater than for Category 1 species and therefore species in Category 2 are still considered at high risk (Fig. 1 and Fig. 3). Although a high proportion of species are predicted to be exposed to climate change, sensitivity was low for many species if suitable habitat was still available or even increased overall (Category 3, Fig. 4c).

### Dispersal Barriers

By assuming an increased cost of dispersal across the open sea, predicted suitable habitat in Tasmania was removed for species currently found only on the mainland. Potentially suitable habitat could be available in Tasmania for up to 73 new species by 2085 under the RCP8.5 scenario (Fig. 4a and c). In many cases losing this potential dispersal option was not significant, but for 24 species the increase in the sensitivity weighting was sufficient to alter their overall score and switch the category of vulnerability from Category 3 to Category 1 or 2 (see Fig. 1). Changing the nature of the Bass Strait to a dispersal barrier is particularly significant for the projections for three upland specialists found on the mainland (*Cordulephya montana* Tillyard 1911, *Austroaeschna subapicalis* Theischinger 1982, *A. flavomaculata* Tillyard 1916), reducing the availability of their potential new habitat by 36–90%.

### Conservation Priorities for Vulnerable Species

**Figure 5:**
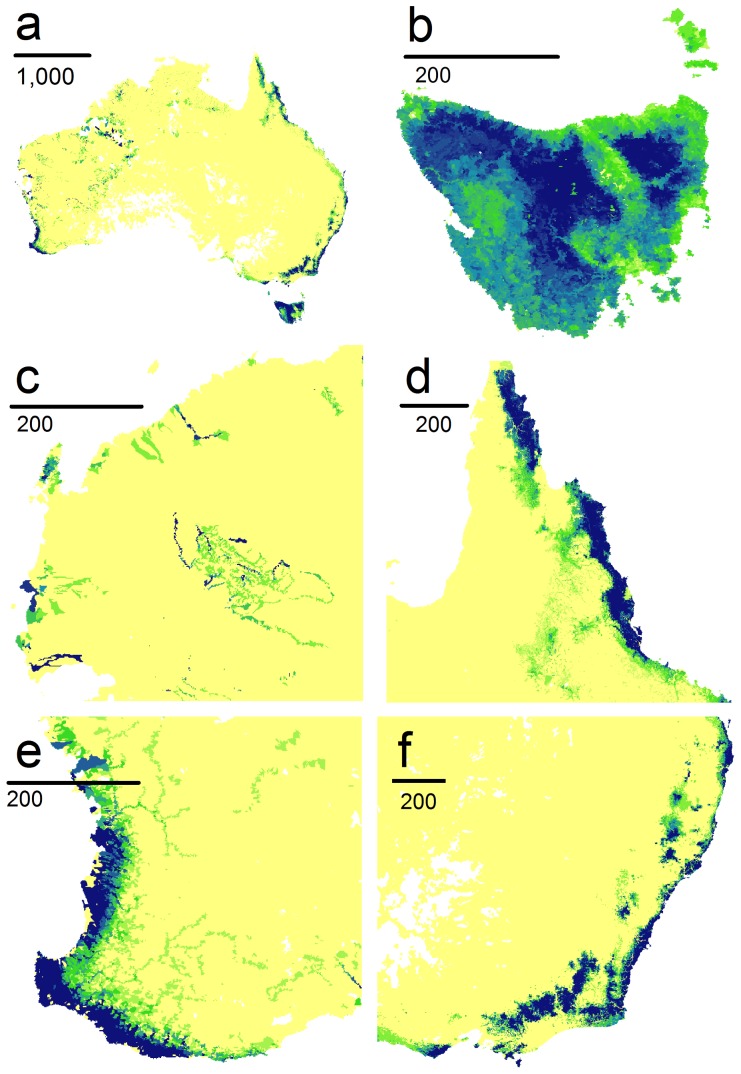
Map of conservation priorities for Odonata vulnerable to climate change in Categories 1 and 2. The panels show priorities in dark blue for (a) Australia, and regional views of (b) Tasmania, (c) the north-west, (d) Cape York peninsula, (e) the south-west and (f) the south-east.

Priority streams and rivers important for conserving the highly vulnerable Category 1 and high risk Category 2 species varied for different time periods and emissions scenarios modelled, but were largely nested within the same core regions (Fig. 5). For the most vulnerable Category 1 species, pockets of permanent water in the Pilbara and north-west of Australia are critical, in particular the Gascoyne and Ashburton rivers. By 2085, there is also a strong emphasis on coastal New South Wales and high altitude areas extending south to the Australian Alps. Without assuming high dispersal limitations to crossing the Bass Strait, Tasmania will also be an important conservation focus. Although not under pressure to disperse, Category 2 species would become increasingly restricted to pockets of suitable habitat within the Wet Tropics and east Cape York peninsula in northern Queensland, the far south-west of Western Australia, Tasmania and small areas within the Kimberley in the north.

## Discussion

This study predicts that 56–69% (153–187 species) of the Australian Odonata modelled will experience a decline in habitat extent by 2085 as a result of climate change, including a number of potential extinctions in the medium and long term. A third of modelled species were considered highly vulnerable or vulnerable by 2085–RCP8.5 (Category 1 and 2) and though species vulnerability was reduced under a more moderate emissions scenario (RCP6) they remain highly dependent on their ability to rapidly track shifting habitats. Priorities for the conservation of vulnerable species are highest in the south-west and south east of the continent, the Wet Tropics region, and in the rivers in the north-west.

Species classified as uncommon prior to modelling were more likely to be vulnerable to the effects of climate change because of their higher exposure. This seems reasonable given stochastic fluctuations from climatic disturbances represent a greater risk to small populations [39]. Australian Odonata appear to face a similar degree of threat as European aquatic macroinvertebrates where 57% of species are predicted to decline by 2080 [16]. Several recent modelling studies have assessed climate change effects on a variety of taxa across continental Australia [45], [46], [88] and Odonata appear to be among the less threatened taxa, although the rarest taxa were not modelled. Although there is some congruence between the distribution of the most vulnerable Odonata and species of birds and crayfish, differences in the distribution of threatened terrestrial and freshwater taxa demonstrates the importance of combining datasets to avoid taxonomic biases when setting conservation priorities [89].

### Modelling limitations

All models could be improved with greater availability of occurrence records [18], or more detailed environmental data [90]. However, the main cause of uncertainty stems from the fact that modelling techniques that make projections based on environmental predictors and presence-only data are at risk of over-estimating suitable habitat extent and including errors of commission because the models assume that all suitable climate space is occupied [91]. Although we account for a number of issues including testing and incorporating a number of non-climatic variables, targeting selection of background points and limiting the degree of extrapolation to novel environments [22], other factors including local habitat conditions, dispersal and species interactions could limit species occurrence within regions of environmental suitability.

While climate and historical factors account for the distribution of freshwater biota at regional spatial scales [92], and the high spatial resolution of the study increased the potential for microclimatic refugia to be identified [91], [93], species occurrence within stream segments is often determined by additional factors such as water volume, habitat heterogeneity, water chemistry, temperature, disturbance and predation e.g. [94], [95]. If these conditions are not suitable within a climatically suitable region [96], then by default a species will be absent from the entire region. For example, the extent of stream habitat available to species specialising in riffles (e.g. *Lestoideidae* spp.), bogs (e.g. *Petalura* spp.) or waterfalls (e.g. *Austropetalia* spp.) will only be a fraction of the subcatchment. In addition, an important factor affecting habitat suitability is human disturbance, with large areas of the landscape already modified [97]. Highly disturbed sites could have been excluded in this analysis, except that our understanding of how rapidly habitat suitability changes, and at what point this could exclude a species, is poor. One method for improving our understanding would be to examine the assembly rules that determine local composition from a species pool generated by SDMs [98].

Although we include changes in stream hydrology within our models, climate change could alter the intensity of cease-to-flow events, floods, droughts and increase evaporation of pool habitats, modifying the true nature of habitat availability within a subcatchment from year to year [32], [99]. A switch from perennial to intermittent streams and ponds reduces the time available for larvae to complete development, but may well suit some taxa such as Lestidae [38]. The threat of saltwater intrusion as a result of sea-level rise is also potentially under-appreciated, as many species were projected to lose habitat along the east coast, including some dune system specialists [100], [101]. Finally, it is worth noting that a species may persist in a region modelled as climatically unsuitable. *Nososticta pilbara* was predicted to lose all climatically suitable habitat, but because it primarily occurs in a few groundwater-fed streams, it may persist in these refuge habitats in the future, resilient to the broader changes in climate [102].

For suitable habitat to support a particular species it must also be within dispersal range. Odonata are among the strongest of flying insects, but dispersal ability can still limit their ability to colonise suitable habitat e.g. [103]. Estimates of dispersal ability could be improved through more intensive monitoring of range shifts or by mark-recapture studies [104], but even then it can be difficult to relate species’ traits and landscape suitability to the distances travelled in response to climate change [87].

Our assessment of the contribution dispersal could have to vulnerability is more thorough than previous studies that have simply assumed either full- and no-dispersal [105]. Some species modelled in this study will not disperse as quickly as 15 km year^−1^, perhaps because they have multi-year development as larvae [83], or due to preferences for lentic or lotic habitats [106]. The Bass Strait is likely to present a dispersal barrier to at least some of the 24 species we predict will be affected, and exacerbate the decline in available habitat. Furthermore, while many species classified in Category 3 are not considered at risk because their sensitivity is low, this will only be the case if they can colonise new habitats, and their progress should be monitored.

Although Odonata are generalist predators, and therefore not reliant on particular prey species, competition amongst ecologically similar species or with other macroinvertebrates could also modify their future distributions. For example, the competitive balance between two coexisting dragonfly species in Germany is predicted to become skewed as temperature increases because one will grow faster, and is subsequently more likely to prey on the smaller conspecific larvae [107]. Changes in the structure of fish assemblages [26] could also result in changes to predation pressure [108].

Based on the range of limitations that could potentially reduce the realised distribution of species from the modelled extent, the suitability scores are best viewed as a species’ maximum potential abundance in an area [109]. Therefore, although some species may adapt or have the flexibility to occupy novel climates, the risk of local and potentially global extinction is likely to be significantly higher than we can currently identify due to our limited knowledge of species ecology [19]. Furthermore, insufficient records for 51 Odonata meant SDMs could not be applied to the species potentially at greatest risk under climate change.

### Implications for Management and Conservation

Australia’s low relief offers little capacity for altitudinal movement, meaning most species must undergo latitudinal shifts to stay within their current environmental envelopes. All species determined to be vulnerable or highly vulnerable are endemic to Australia, and given Australia’s history of isolation from neighbouring countries such as Papua New Guinea [110], it is unlikely species would be able to reach suitable habitats outside Australia (but see [111]). Our modelling indicated that suitable odonate habitats retreated to higher elevations in the Wet Tropics, where changes in precipitation and cloud cover that threaten rainforest vertebrates could also affect these invertebrates [112]. Several high elevation species in New South Wales are also highly isolated, and these regions will also become priorities for other species as climate change intensifies. Within cooler region such as Tasmania, regional endemics may persist unless other environmental changes alter habitat suitability e.g. fire [113], and if they are not competitively displaced by immigrant species [107]. Many species endemic to the Pilbara or south-west Australia will be reliant on the availability of permanent freshwater to avoid extinction [102], [114].

Preventing the loss of species in the face of multiple stressors, many of which are synergistic with the effects of climate change, is a virtually impossible task [115]. Nonetheless, climate change presents a clear danger to Odonata and other freshwater species and we can improve conservation efficiency by incorporating these projections into decision-making [116], identifying suitable strategies before declines become severe [117]. Habitat restoration can be effective at local scales and insect populations including Odonata can be quick to respond [118], [119], although problems may persist if restoration does not account for upstream influences or when sites are isolated [120]. Freshwater refugia will be crucial to species persistence in regions like the Wet Tropics [45] and the Australian alpine region, but also more generally during droughts, as the climate continues to change [121].

Although the predicted risks to Australian Odonata from climate change outlined in this study are significant, they are probably quite conservative. Other threats such as habitat modification and water extraction would also need to be included to avoid underestimating the true extinction risk [122]. Shifts in suitable habitat predicted by this analysis could soon become observed range shifts and the current and future value of streams should be considered in conservation planning.

## Supporting Information

Table S1 **Sources of Australian Odonata Occurrence Data.**
(PDF)

Table S2 **Ensemble model scores and scenario range changes.**
(PDF)

File S1 **Predictor variables and model validation summary.** This file contains Table A and Figure S1. Table A, Environmental factors used to predict species distributions and their mean predictive importance (r2). Figure S1, The ensemble model score for each species (n = 270) based on either the area under the receiver operating characteristic curve (AUC), or the True Skill Statistic (TSS).
(PDF)

File S2 **Demonstration of species vulnerability assessment.** This document contains Figure S2–S10 and Table S3–S7. Figure S2, Distribution of occurrence records for *Notoaeschna sagittata* (green, n = 336), *Tetrathemis irregularis cladophila* (red, n = 57) and *Austrosticta frater* (blue, n = 24). Figure S3, Predicted habitat suitability for *Notoaeschna sagittata* under current and future climatic conditions. Figure S4, Predicted habitat suitability for *Tetrathemis irregularis cladophila* under current and future climatic conditions. Figure S5, Predicted habitat suitability for *Austrosticta frater* under current and future climatic conditions. Figure S6, Density plot showing the frequency of habitat suitability scores within the current and future range of *Tetrathemis irregularis cladophila.* Figure S7, Distance (km) from existing records of a species to all other sites. Figure S8, The probability of dispersal according to distance from occurrence records. Figure S9, Sensitivity weighting for RCP8.5 2085 plotted against dispersal threshold. Figure S10, Categories of vulnerability to climate change effects for species based on three components; exposure, sensitivity and dispersal pressure. Table S3, Predicted change in suitable habitat for two emissions scenarios for 2055 and 2085. Table S4, Summary of scoring system for environmental exposure. Table S5, Environmental exposure scores for *Notoaeschna sagittata, Tetrathemis irregularis cladophila* and *Austrosticta frater*. Table S6, Average habitat shifts (km) under climate change scenarios. Table S7, Scoring for the dispersal component of the vulnerability assessment.
(PDF)
