# Supplementary material for: Continental-Scale Assessment of Risk to the Australian Odonata from Climate Change
Source: PLoS One. 2014 Feb 13;9(2):e88958. doi: 10.1371/journal.pone.0088958 (PMC3923880; doi:10.1371/journal.pone.0088958)
Supplement: Table S1 — Sources of Australian Odonata Occurrence Data. (PDF) [file pone.0088958.s001.pdf]

Table S1 Sources of Australian Odonata Data

Source	Approximate # Records
**Museums**	**29,400**
Australian Museum	1600
Australian National Insect Collection	16100
National Museum of Victoria	650
Northern Territory Museum	850
Queensland Museum	8600
Queen Victoria Museum and Art Gallery	350
South Australian Museum	1000
Tasmanian Museum and Art Gallery	150
Western Australian Museum	100
**Government**	**15,450**
Australian Plant Pest Database	1500
Bush Blitz (see http://www.bushblitz.org.au/)	100
Georges River Catchment Authority	200
Sydney Catchment Authority	100
New South Wales Office of Environment and Heritage	7450
Queensland Department of Environment and Resource Management	200
Department of Environment and Primary Industries	3100
Western Australia Department of Environment and Conservation	2800
**Private**	**13,300**
Dennis Paulson	1450
Ian Endersby	450
Roger Garrison	350
Vincent Kalkman	400
Karen Sutcliffe (PhD thesis)	1500
Nick Donnely	450
Fons Peels	450
Reiner Richter	3100
Gunther Theischinger	5150
