# Supplementary material for: Continental-Scale Assessment of Risk to the Australian Odonata from Climate Change
Source: PLoS One. 2014 Feb 13;9(2):e88958. doi: 10.1371/journal.pone.0088958 (PMC3923880; doi:10.1371/journal.pone.0088958)
Supplement: Table S2 — Ensemble model scores and scenario range changes. (PDF) [file pone.0088958.s002.pdf]

Table S2. Model predictions for all species under emissions scenarion RCP8.5. The region code and record group dictate which group of variables were used to predict species habitat suitability (C=continental, N=north, NE=north-east, E=east, S=south and W=west). Model scores are for the True Skills Statistic (TSS) and area under the operating curve (AUC). Current range is the current extent of suitable habitat in km2.

For projections in 2055 and 2085 values indicate the percentage loss (Loss) and percentage (gain) in suitable habitat extent, the sum of the climate exposure score (C), whether the senstivity score was above one and considered vulnerable (S), the sum of dispersal score measurements (D), and the overall vulnerability class (V). Vulerability classes; vulnerable across all categories(1), combination of exposure and sensitivity (2), exposure and dispersal (3), dispersal and sensitivity(4), exposure only (5), sensitivity only (6), dispersal only (7) and not vulnerable for any category (0).

Species	Region	Record Group	Model Score		Current Range	RCP85_2055						RCP85_2085					
			TSS	AUC		Loss	Gain	C	S	D	V	Loss	Gain	C	S	D	V
Acanthaeschna victoria Martin, 1901	E	U	0.952	0.993	15129	40	176	0.5		1	0	57	170	3		5	3
Aciagrion fragile (Tillyard, 1906)	NE	C	0.821	0.954	109898	38	19	1.5		1	5	70	4	1.5	Y	1	2
Adversaeschna brevistyla (Rambur, 1842)	C	C	0.72	0.811	891865	33	3	0.5		1	0	45	5	1.5		1	5
Aethriamanta circumsignata Selys, 1897	NE	C	0.801	0.944	178981	27	90	1		1	5	44	41	1.5		1	5
Aethriamanta nymphaeae Lieftinck, 1949	NE	U	0.883	0.975	38953	36	166	1		1	5	51	160	2.5		1	5
Agriocnemis argentea Tillyard, 1906	N	C	0.81	0.951	287560	66	32	1.5		1	5	78	41	2		1	5
Agriocnemis dobsoni Fraser, 1954	NE	U	0.947	0.994	212	100	41	1	Y	4	1	100	8	2.5	Y	4	1
Agriocnemis kunjina Watson, 1969	W	U	0.942	0.985	5291	100	41	1	Y	5	1	100	41	1.5	Y	5	1
Agriocnemis pygmaea (Rambur, 1842)	NE	C	0.691	0.864	316948	36	140	1		1	5	52	35	1		1	5
Agriocnemis rubricauda Tillyard, 1913	NE	C	0.794	0.956	62119	67	17	1.5		0	5	93	14	3	Y	3	1
Agrionoptera insignis allogenes Tillyard, 1908	NE	C	0.883	0.997	99676	32	22	1		1	5	43	16	2.5		1	5
Agrionoptera longitudinalis biserialis Selys, 1879	NE	C	0.93	0.968	5571	34	79	1		1	5	65	71	1.5		1	5
Anaciaeschna jaspidea (Burmeister, 1839)	N	U	0.951	0.995	18778	27	125	1		1	5	50	104	3		1	5
Anax gibbosulus Rambur, 1842	NE	C	0.859	0.958	123661	21	149	1.5		1	5	38	53	2.5		1	5
Anax guttatus (Burmeister, 1839)	N	C	0.855	1.009	159657	12	136	1.5		1	5	24	128	3		1	5
Anax papuensis (Burmeister, 1839)	C	C	0.663	0.777	6137882	1	0	1		1	5	3	1	1		0	5
Antipodogomphus acolythus (Martin, 1901)	E	C	0.846	0.969	246243	31	102	1		1	5	30	110	2		1	5
Antipodogomphus hodgkini Watson, 1969	W	U	0.939	0.984	1711	100	34	2		4	3	100	55	2		3	3
Antipodogomphus proselythus (Martin, 1901)	NE	U	0.952	0.988	13345	64	160	1.5		1	5	84	185	2.5		2	5
Antipodophlebia asthenes (Tillyard, 1916)	E	U	0.91	0.983	34641	54	65	1.5		1	5	80	37	2.5	Y	5	1
Apocordulia macrops Watson, 1980	S	C	0.953	0.991	95762	40	51	1.5		1	5	60	46	2		1	5

Species	Region	Record Group	Model Score		Current Range	RCP85_2055						RCP85_2085					
			TSS	AUC		Loss	Gain	C	S	D	V	Loss	Gain	C	S	D	V
Archaeophya adamsi Fraser, 1959	E	U	0.926	0.97	4338	98	144	2.5		3	3	100	45	3	Y	5	1
Archaeophya magnifica Theischinger & Watson, 1978	NE	U	0.945	0.987	7772	40	60	1		1	5	64	41	2		0	5
Archaeosynthemis leachii (Selys, 1871)	W	C	0.899	0.979	25874	42	1	1		1	5	61	3	2	Y	1	2
Archaeosynthemis occidentalis (Tillyard, 1910)	W	C	0.905	0.976	75771	16	4	1		0	5	27	7	2	Y	1	2
Archaeosynthemis orientalis (Tillyard, 1910)	S	C	0.811	0.944	111703	42	22	0		1	0	65	24	1.5		1	5
Archaeosynthemis spiniger (Tillyard, 1913)	W	U	0.949	0.986	24167	62	38	2	Y	0	2	86	14	2	Y	0	2
Archiargiolestes parvulus (Watson, 1977)	W	U	0.943	0.99	3274	98	91	2.5		1	5	100	41	4.5		2	5
Archiargiolestes pusillissimus Kennedy, 1925	W	U	0.953	0.992	20201	69	8	1.5	Y	1	2	72	16	1.5		1	5
Archiargiolestes pusillus (Tillyard, 1908)	W	C	0.856	0.963	96223	31	8	0.5		1	0	42	10	1.5	Y	1	2
Archibasis mimetes (Tillyard, 1913)	NE	C	0.938	0.986	15753	42	32	1		0	5	65	25	2		0	5
Archipetalia auriculata Tillyard, 1917	S	U	0.921	0.979	6212	94	4	2	Y	3	1	100	0	3	Y	5	1
Argiocnemis rubescens Selys, 1877	C	C	0.701	0.869	590293	37	169	1		1	5	60	161	1.5		2	5
Armagomphus armiger (Tillyard, 1913)	W	U	0.952	0.993	6818	100	0	2	Y	5	1	100	0	2	Y	5	1
Austroaeschna anacantha Tillyard, 1908	W	C	0.861	0.966	19345	50	0	2	Y	0	2	69	0	2	Y	1	2
Austroaeschna atrata Martin, 1901	S	C	0.86	0.974	44034	44	50	1		1	5	62	55	2		5	3
Austroaeschna cooloola Theischinger, 1991	E	U	0.951	0.998	479	100	84	3		2	5	100	73	4		5	3
Austroaeschna flavomaculata Tillyard, 1916	S	C	0.949	0.991	13195	54	151	0.5		0	0	75	92	2		5	3
Austroaeschna hardyi Tillyard 1907	S	C	0.71	0.894	28494	33	13	0.5		1	0	48	13	2		1	5
Austroaeschna inermis Martin, 1901	S	C	0.808	0.958	136222	39	25	1		3	3	66	29	1.5	Y	3	1
Austroaeschna ingrid Theischinger, 2008	S	U	0.923	0.968	907	100	0	2.5	Y	5	1	100	0	3	Y	5	1
Austroaeschna muelleri Theischinger, 1982	E	U	0.926	0.972	200	100	89	4	Y	5	1	100	0	4	Y	5	1
Austroaeschna multipunctata (Martin, 1901)	S	C	0.748	0.95	205204	38	33	0.5		1	0	62	30	0.5		4	7
Austroaeschna obscura Theischinger, 1982	S	C	0.876	0.966	33824	84	110	2.5		4	3	94	122	2.5		4	3
Austroaeschna parvistigma (Selys, 1883)	S	C	0.719	0.926	342452	41	0	0		0	0	61	0	1.5	Y	0	2
Austroaeschna pinheyi Theischinger, 2001	E	C	0.951	0.997	1886	100	351	3	Y	5	1	100	187	3		5	3
Austroaeschna pulchra Tillyard, 1909	S	C	0.682	0.905	271809	17	22	1		1	5	32	23	2		2	5
Austroaeschna sigma Theischinger, 1982	E	C	0.803	0.949	118725	26	123	1		1	5	43	131	2.5		2	5
Austroaeschna speciosa Sjöstedt, 1917	NE	U	0.951	0.996	6797	73	1	1	Y	0	2	93	0	1.5	Y	0	2
Austroaeschna subapicalis Theischinger, 1982	S	C	0.787	0.972	59114	47	67	1		5	3	69	72	1.5		4	3
Austroaeschna tasmanica Tillyard, 1916	S	C	0.863	0.976	18344	35	28	0		1	0	64	33	1.5		1	5
Austroaeschna unicornis (Martin, 1901)	S	C	0.767	0.919	447473	28	5	0.5		1	0	43	4	1.5		0	5

Species	Region	Record Group	Model Score		Current Range	RCP85_2055						RCP85_2085					
			TSS	AUC		Loss	Gain	C	S	D	V	Loss	Gain	C	S	D	V
Austroagrion cyane (Selys, 1876)	W	C	0.869	0.991	513930	39	77	0		1	0	43	85	0.5		1	0
Austroagrion exclamationis Campion, 1915	NE	C	0.722	0.947	204037	43	65	1.5		1	5	86	14	2	Y	1	2
Austroagrion pindrina Watson, 1969	W	C	0.867	0.969	143842	89	3	2		3	3	89	3	2	Y	2	2
Austroagrion watsoni Lieftinck, 1982	C	C	0.704	0.812	979482	42	15	0.5		1	0	51	15	1		0	5
Austroargiolestes alpinus (Tillyard, 1913)	S	U	0.947	0.994	473	8	67	2.5		1	5	100	0	2.5	Y	0	2
Austroargiolestes amabilis (Förster, 1899)	E	C	0.832	0.955	54161	52	48	1		1	5	90	23	2.5	Y	5	1
Austroargiolestes aureus (Tillyard, 1906)	NE	C	0.936	0.983	9857	46	15	1		1	5	66	2	2	Y	0	2
Austroargiolestes brookhousei Theischinger & O'Farrell, 1986	S	U	0.92	0.961	2046	73	258	1.5		5	3	87	105	2.5		5	3
Austroargiolestes calcaris (Fraser, 1958)	S	C	0.826	0.96	84490	46	43	0.5		1	0	66	49	1.5		4	3
Austroargiolestes christine Theischinger & O'Farrell, 1986	E	C	0.916	0.956	3897	47	335	2		1	5	90	18	2	Y	5	1
Austroargiolestes chrysoides (Tillyard, 1913)	E	C	0.921	0.961	3915	87	254	2.5		5	3	98	224	3		4	3
Austroargiolestes icteromelas (Selys, 1862)	E	C	0.682	0.851	481535	25	15	1		1	5	38	17	1.5		2	5
Austroargiolestes isabellae Theischinger & O'Farrell, 1986	E	C	0.944	0.984	15575	81	153	2.5		4	3	98	179	2.5		3	3
Austrocnemis maccullochi (Tillyard, 1926)	N	C	0.915	0.972	33091	39	516	2		1	5	66	142	3		2	5
Austrocnemis splendida (Martin, 1901)	E	C	0.745	0.938	421876	35	44	1		1	5	46	50	1.5		1	5
Austrocordulia leonardi Theischinger, 1973	E	U	0.933	0.98	707	100	152	3	Y	4	1	100	318	3		5	3
Austrocordulia refracta Tillyard, 1909	E	C	0.727	0.92	298017	32	32	1		1	5	46	36	1.5		1	5
Austroepigomphus gordoni (Watson, 1962)	W	C	0.813	0.957	520849	70	20	1.5	Y	4	1	74	26	2	Y	3	1
Austroepigomphus praeruptus (Selys, 1857)	E	C	0.771	0.963	370755	49	37	2		1	5	66	49	2		1	5
Austroepigomphus turneri (Martin, 1901)	NE	C	0.839	0.959	267132	44	66	1.5		1	5	79	24	1.5	Y	3	1
Austrogomphus amphiclitus (Selys, 1873)	E	C	0.706	0.927	247376	39	52	1		1	5	56	67	2		2	5
Austrogomphus arbustorum Tillyard, 1906	NE	U	0.952	0.998	8941	61	229	2.5		1	5	75	269	2.5		1	5
Austrogomphus australis Dale, 1854	C	C	0.696	0.94	814103	63	20	1.5		1	5	68	22	2.5		1	5
Austrogomphus bifurcatus Tillyard, 1909	NE	U	0.937	0.984	9998	40	21	1		1	5	68	13	2	Y	0	2
Austrogomphus collaris Hagen, 1854	W	C	0.855	0.972	52315	37	24	0.5		1	0	59	11	1.5		0	5
Austrogomphus cornutus Watson, 1991	E	C	0.722	0.92	690334	35	15	1		0	5	51	17	2		1	5
Austrogomphus divaricatus Watson, 1991	NE	C	0.917	0.986	19346	51	17	0.5		1	0	76	6	1.5	Y	1	2
Austrogomphus guerini (Rambur, 1842)	S	C	0.748	0.906	262475	47	6	1		0	5	69	8	1.5	Y	1	2
Austrogomphus mjobergi Sjöstedt, 1917	N	C	0.924	0.986	101458	61	724	2		1	5	85	538	2		4	3
Austrogomphus ochraceus (Selys, 1869)	E	C	0.709	0.889	325917	27	32	1		1	5	43	33	2		2	5
Austrogomphus prasinus Tillyard, 1906	NE	C	0.893	0.969	19830	64	15	0.5		0	0	89	17	1.5	Y	0	2

Species	Region	Record Group	Model Score		Current Range	RCP85_2055						RCP85_2085					
			TSS	AUC		Loss	Gain	C	S	D	V	Loss	Gain	C	S	D	V
Austrogynacantha heterogena Tillyard, 1908	C	C	0.747	0.891	1471593	29	63	1		1	5	34	57	1.5		1	5
Austrolestes aleison Watson & Moulds, 1979	W	C	0.918	0.984	36915	27	40	0.5		1	0	70	45	1.5		1	5
Austrolestes analis (Rambur, 1842)	C	C	0.729	0.847	958274	34	8	0		1	0	44	8	1.5		1	5
Austrolestes annulosus (Selys, 1862)	C	C	0.736	0.942	804125	31	3	0		0	0	40	3	0.5		0	0
Austrolestes aridus (Tillyard, 1908)	C	C	0.663	0.91	3139891	68	5	1	Y	2	2	72	7	1.5	Y	0	2
Austrolestes cingulatus (Burmeister, 1839)	S	C	0.74	0.94	321128	38	3	0.5	Y	0	6	62	5	1.5	Y	1	2
Austrolestes insularis Tillyard, 1913	N	C	0.793	0.93	225815	41	36	1.5		1	5	78	17	2	Y	1	2
Austrolestes io (Selys, 1862)	C	C	0.85	0.977	463180	25	26	0		1	0	34	25	0.5		1	0
Austrolestes leda (Selys, 1862)	C	C	0.695	0.812	958761	38	12	0.5		1	0	48	12	2		1	5
Austrolestes minjerriba Watson, 1979	E	C	0.926	0.969	4832	95	56	1		4	3	100	39	2.5	Y	4	1
Austrolestes psyche (Hagen, 1862)	S	C	0.748	0.894	485223	34	2	0.5		0	0	52	3	2	Y	0	2
Austropetalia patricia (Tillyard, 1910)	S	U	0.924	0.97	585	100	533	2.5		5	3	100	300	3		4	3
Austropetalia tonyana Theischinger, 1995	S	U	0.862	0.964	54273	55	36	1		0	5	69	26	2	Y	4	1
Austrophlebia costalis (Tillyard, 1907)	E	C	0.824	0.964	73920	29	88	1.5		1	5	57	109	2		2	5
Austrophlebia subcostalis Theischinger, 1996	NE	U	0.93	0.971	2449	80	31	1		0	5	100	0	2	Y	5	1
Austrophya mystica Tillyard, 1909	NE	U	0.907	0.983	14427	73	42	1		4	3	82	7	1.5	Y	1	2
Austrosticta fieldi Tillyard, 1908	N	U	0.952	0.993	60927	71	80	2		3	3	94	56	2		5	3
Austrosticta frater Theischinger, 1997	N	U	0.952	0.995	23119	64	284	2		1	5	98	186	3		3	3
Austrosynthemis cyanitincta (Tillyard, 1908)	W	C	0.897	0.978	26641	53	0	2	Y	2	2	76	1	2	Y	1	2
Austrothemis nigrescens (Martin, 1901)	C	C	0.802	0.955	335342	24	60	0		1	0	27	66	1		1	5
Brachydiplax denticauda (Brauer, 1867)	NE	C	0.778	0.951	124617	26	155	1		1	5	49	47	2.5		1	5
Brachydiplax duivenbodei (Brauer, 1866)	NE	U	0.951	0.995	8583	19	178	1.5		1	5	24	318	3		2	5
Caliagrion billinghursti (Martin, 1901)	S	C	0.892	0.977	77444	63	98	2		1	5	76	146	2.5		2	5
Camacinia othello Tillyard, 1908	NE	U	0.93	0.976	2993	21	301	1		1	5	47	555	3		1	5
Ceriagrion aeruginosum (Brauer, 1869)	NE	C	0.719	0.964	345650	14	56	1		1	5	35	21	1.5		1	5
Chorismagrion risi Morton, 1914	NE	C	0.922	0.957	6335	54	74	1		2	5	81	39	2		1	5
Choristhemis flavoterminata (Martin, 1901)	E	C	0.715	0.917	329891	30	48	0.5		1	0	43	57	1.5		2	5
Coenagrion lyelli (Tillyard, 1913)	S	C	0.818	0.962	144651	74	1	0.5	Y	0	6	87	1	1	Y	0	2
Cordulephya montana Tillyard, 1911	S	U	0.936	0.979	30014	78	55	1	Y	3	1	90	43	2	Y	5	1
Cordulephya pygmaea Selys, 1870	E	C	0.698	0.918	252285	30	33	1		0	5	47	34	2		1	5
Crocothemis nigrifrons (Kirby, 1894)	C	C	0.732	0.881	983366	25	92	1		1	5	24	89	1.5		1	5

Species	Region	Record Group	Model Score		Current Range	RCP85_2055						RCP85_2085					
			TSS	AUC		Loss	Gain	C	S	D	V	Loss	Gain	C	S	D	V
Dendroaeschna conspersa (Tillyard, 1907)	E	C	0.828	0.987	54787	44	80	1.5		1	5	66	96	2.5		2	5
Diphlebia coerulescens Tillyard, 1913	E	C	0.878	0.972	27169	46	93	1.5		1	5	82	109	2.5		3	3
Diphlebia euphoeoides Tillyard, 1907	NE	C	0.875	0.964	20331	54	9	0.5		1	0	77	4	1.5	Y	1	2
Diphlebia hybridoides Tillyard, 1912	NE	U	0.931	0.972	4869	70	26	1		1	5	84	11	2	Y	1	2
Diphlebia lestoides (Selys, 1853)	S	C	0.71	0.909	252136	31	40	1		1	5	52	44	2		2	5
Diphlebia nymphoides Tillyard, 1912	S	C	0.714	0.914	288748	34	34	1		0	5	48	32	2		1	5
Diplacodes bipunctata (Brauer, 1865)	C	C	0.649	0.704	6134082	1	1	1		1	5	3	1	1		1	5
Diplacodes haematodes (Burmeister, 1839)	C	C	0.748	0.795	5041586	3	10	1		1	5	4	14	1.5		1	5
Diplacodes melanopsis (Martin, 1901)	E	C	0.723	0.934	280866	31	36	0.5		1	0	48	40	2		1	5
Diplacodes nebulosa (Fabricius, 1793)	N	C	0.811	0.946	96726	16	125	1.5		1	5	29	41	2		1	5
Diplacodes trivialis (Rambur, 1842)	NE	C	0.726	0.931	189245	21	186	1		1	5	59	38	2.5		1	5
Dromaeschna forcipata (Tillyard, 1907)	NE	C	0.907	0.974	15888	33	16	0.5		0	0	60	4	1.5	Y	1	2
Dromaeschna weiskei (Förster, 1908)	NE	C	0.919	0.985	10057	41	9	1		0	5	74	1	2	Y	0	2
Episynlestes albicauda (Tillyard, 1913)	E	C	0.819	0.968	80800	79	38	1.5		4	3	95	26	2.5	Y	4	1
Episynlestes cristatus Watson & Moulds, 1977	NE	U	0.92	0.965	1402	92	71	1		1	5	100	1	2	Y	3	1
Eurysticta coolawanyah Watson, 1969	W	C	0.865	0.969	298177	83	33	2	Y	5	1	84	40	2	Y	4	1
Eusynthemis aurolineata (Tillyard, 1913)	E	C	0.809	0.944	78530	34	146	1.5		1	5	64	116	2.5		4	3
Eusynthemis brevistyla (Selys, 1871)	S	C	0.764	0.961	223119	35	32	1		1	5	53	32	1.5		2	5
Eusynthemis guttata (Selys, 1871)	S	C	0.785	0.952	116988	46	51	0.5		2	0	68	49	1.5		4	3
Eusynthemis nigra (Tillyard, 1906)	E	C	0.79	0.947	107382	22	86	0		1	0	41	109	1.5		1	5
Eusynthemis rentziana Theischinger, 1998	E	U	0.896	0.977	32567	74	34	1.5	Y	4	1	94	16	2.5	Y	5	1
Eusynthemis tillyardi Theischinger, 1995	S	C	0.814	0.949	102556	52	29	1.5		1	5	62	26	2		1	5
Eusynthemis ursula Theischinger, 1998	S	U	0.946	0.993	223	77	70	2		1	5	100	0	3	Y	5	1
Eusynthemis virgula (Selys, 1874)	S	C	0.746	0.978	211123	40	27	1		1	5	63	22	2		4	3
Griseargiolestes albescens (Tillyard, 1913)	E	C	0.954	0.995	7875	95	43	2		5	3	100	38	3	Y	4	1
Griseargiolestes bucki Theischinger, 1998	E	U	0.924	0.967	2744	88	290	1.5	Y	3	1	93	131	2.5		5	3
Griseargiolestes eboracus (Tillyard, 1913)	E	C	0.844	0.965	100286	53	46	1		1	5	83	36	1.5	Y	5	1
Griseargiolestes fontanus (Tillyard, 1913)	E	U	0.952	0.989	9302	97	9	2	Y	4	1	100	4	2.5	Y	5	1
Griseargiolestes griseus (Hagen, 1862)	S	C	0.849	1	126301	46	35	1		1	5	60	33	2		2	5
Griseargiolestes intermedius (Tillyard, 1913)	S	C	0.911	0.97	28031	37	56	1		0	5	56	37	2		5	3
Griseargiolestes metallicus (Sjöstedt, 1917)	NE	U	0.922	0.966	845	100	9	2	Y	3	1	100	0	2	Y	5	1

Species	Region	Record Group	Model Score		Current Range	RCP85_2055						RCP85_2085					
			TSS	AUC		Loss	Gain	C	S	D	V	Loss	Gain	C	S	D	V
Gynacantha dobsoni Fraser, 1951	N	C	0.804	0.946	88543	38	282	1.5		1	5	61	71	1.5		1	5
Gynacantha mocsaryi Förster, 1898	NE	C	0.935	0.988	13843	22	53	1		1	5	51	36	3.5		0	5
Gynacantha nourlangie Theischinger & Watson, 1991	N	C	0.78	0.97	166293	41	120	2		1	5	91	28	2	Y	3	1
Gynacantha rosenbergi Kaup, 1867	NE	C	0.927	0.989	12310	34	43	1		1	5	60	45	1.5		1	5
Hemicordulia australiae (Rambur, 1842)	C	C	0.692	0.856	557063	32	26	1		1	5	37	29	1.5		1	5
Hemicordulia continentalis Martin, 1901	E	C	0.838	0.966	64490	39	28	0.5		1	0	54	22	2.5		1	5
Hemicordulia intermedia (Selys, 1871)	C	C	0.734	0.892	861310	38	66	1		1	5	65	55	1.5		1	5
Hemicordulia koomina Watson, 1969	W	U	0.952	0.986	119416	97	3	2	Y	4	1	99	5	2	Y	4	1
Hemicordulia superba Tillyardi, 1911	E	C	0.947	0.991	101316	50	97	2		1	5	76	119	3		1	5
Hemicordulia tau (Selys, 1871)	C	C	0.765	0.935	1768259	30	66	1		1	5	40	115	1.5		1	5
Hemigomphus comitatus (Tillyard, 1909)	NE	C	0.901	0.969	38306	45	31	0.5		0	0	78	14	2	Y	5	1
Hemigomphus cooloola Watson, 1991	E	U	0.952	0.978	125	100	0	2.5	Y	5	1	100	0	4	Y	5	1
Hemigomphus gouldii (Selys, 1854)	E	C	0.736	0.902	304650	26	17	1		1	5	44	18	2		2	5
Hemigomphus heteroclytus Selys, 1854	E	C	0.702	0.905	188130	37	39	1.5		1	5	58	52	2		1	5
Hemigomphus magela Watson, 1991	N	U	0.935	0.978	2316	97	52	1.5	Y	3	1	100	66	2	Y	2	2
Hemigomphus theischingeri Watson, 1991	NE	U	0.938	0.983	2147	87	66	1.5		1	5	97	32	2	Y	0	2
Hemiphlebia mirabilis Selys, 1869	S	C	0.93	0.99	39071	97	50	1		1	5	100	54	1		1	5
Hesperocordulia berthoudi Tillyard, 1911	W	C	0.91	0.979	9110	86	1	2	Y	2	2	100	0	2	Y	5	1
Hydrobasileus brevistylus (Brauer, 1865)	NE	C	0.79	0.956	105908	46	129	1		1	5	66	36	1.5		1	5
Ictinogomphus australis (Selys, 1873)	NE	C	0.711	0.921	523418	22	183	1		1	5	23	185	1.5		1	5
Ictinogomphus dobsoni (Watson, 1969)	W	C	0.943	0.986	137401	88	13	1.5	Y	4	1	91	14	2	Y	3	1
Indolestes alleni (Tillyard, 1913)	N	U	0.952	0.987	13344	27	60	1.5		1	5	39	73	3		1	5
Indolestes obiri Watson, 1979	N	U	0.934	0.979	2766	100	0	1	Y	5	1	100	0	2	Y	5	1
Indolestes tenuissimus (Tillyard, 1906)	NE	C	0.942	0.988	14448	51	34	1.5		1	5	72	33	3		0	5
Ischnura aurora (Brauer, 1865)	C	C	0.642	0.874	5582244	8	9	1		0	5	8	10	1		1	5
Ischnura heterosticta (Burmeister, 1839)	C	C	0.739	0.957	2321650	25	56	1		1	5	34	75	1		1	5
Ischnura pruinescens (Tillyard, 1906)	NE	C	0.767	0.97	126613	57	82	1		1	5	79	13	1.5	Y	1	2
Labidiosticta vallisi (Fraser, 1955)	E	C	0.892	0.982	72550	28	64	1		1	5	44	93	1.5		2	5
Lathrecista asiatica festa (Selys, 1879)	NE	C	0.815	0.975	84211	34	121	1.5		1	5	65	24	3		1	5
Lathrocordulia metallica Tillyard, 1911	W	C	0.936	0.987	11524	97	0	2	Y	2	2	100	0	2	Y	5	1
Lestes concinnus Hagen, 1862	N	C	0.742	0.909	309099	35	172	1.5		1	5	74	51	2		1	5

Species	Region	Record Group	Model Score		Current Range	RCP85_2055						RCP85_2085					
			TSS	AUC		Loss	Gain	C	S	D	V	Loss	Gain	C	S	D	V
Lestoidea brevicauda Theischinger, 1996	NE	U	0.916	0.961	734	80	72	2		1	5	90	101	3		1	5
Lestoidea conjuncta Tillyard, 1913	NE	C	0.922	0.986	10835	49	28	1		1	5	73	13	2	Y	0	2
Lestoidea lewisiana Theischinger, 1996	NE	U	0.944	0.991	354	100	0	3.5	Y	5	1	100	0	4	Y	5	1
Macrodiplax cora (Kaup, 1867)	C	C	0.737	0.962	260085	41	321	1.5		1	5	73	189	1.5		2	5
Macromia tillyardi Martin, 1906	NE	C	0.909	0.982	104862	63	82	1.5		1	5	77	75	1.5		1	5
Micromidia atrifrons (McLachlan, 1883)	E	C	0.848	0.967	90250	29	39	0.5		1	0	52	45	1.5		1	5
Micromidia convergens Theischinger & Watson, 1978	E	U	0.944	0.991	783	100	503	2.5		4	3	99	369	3		3	3
Miniargiolestes minimus (Tillyard, 1908)	W	C	0.889	0.975	26576	45	6	1	Y	1	2	58	1	2	Y	1	2
Nannodiplax rubra Brauer, 1868	C	C	0.718	0.921	420494	15	37	1		1	5	39	12	1.5		1	5
Nannophlebia eludens Tillyard, 1908	NE	C	0.81	0.942	116902	18	40	1		1	5	42	12	1.5		1	5
Nannophlebia injibandi Watson, 1969	N	C	0.954	0.993	314512	53	140	2		3	3	94	105	2		4	3
Nannophlebia mudginberri Watson & Theischinger, 1991	N	U	0.953	0.99	19412	67	108	2		1	5	85	37	3		1	5
Nannophlebia risi Tillyard, 1913	E	C	0.699	0.918	202605	27	41	1		1	5	45	44	2		1	5
Nannophya australis Brauer, 1865	E	C	0.814	0.983	226849	72	47	1	Y	1	2	86	43	1	Y	5	1
Nannophya dalei (Tillyard, 1908)	S	C	0.816	0.974	194220	47	10	0		1	0	60	8	1	Y	1	2
Nannophya occidentalis (Tillyard, 1908)	W	U	0.953	0.99	19107	42	40	0.5		1	0	52	50	1.5		1	5
Neosticta canescens Tillyard, 1913	E	C	0.838	0.959	91359	19	104	1		1	5	35	129	2		2	5
Neosticta fraseri Watson, 1991	NE	C	0.936	0.989	9483	29	49	1		2	5	52	17	1.5		1	5
Neurothemis oligoneura Brauer, 1867	NE	U	0.952	0.994	8363	66	29	2		4	3	82	39	3		3	3
Neurothemis stigmatizans (Fabricius, 1775)	N	C	0.761	0.956	277758	5	140	1.5		1	5	10	150	2		1	5
Nososticta baroalba Watson & Theischinger, 1984	N	C	0.953	0.994	47516	42	58	1		1	5	44	16	1.5	Y	1	2
Nososticta coelestina (Tillyard, 1906)	N	C	0.827	0.95	83749	46	24	1.5		1	5	59	15	1.5		1	5
Nososticta fraterna (Lieftinck, 1933)	N	C	0.819	0.984	92210	76	65	2		4	3	100	18	2	Y	5	1
Nososticta kalumburu Watson & Theischinger, 1984	N	U	0.951	0.992	17434	83	5	2	Y	4	1	90	4	2	Y	3	1
Nososticta koolpinyah Watson & Theischinger, 1984	N	U	0.948	0.991	1605	100	28	2	Y	4	1	100	0	3	Y	5	1
Nososticta koongarra Watson & Theischinger, 1984	N	U	0.929	0.974	1028	100	176	1		3	3	100	50	1	Y	3	1
Nososticta liveringa Watson & Theischinger, 1984	N	C	0.924	0.972	97573	70	107	2		4	3	92	18	2	Y	5	1
Nososticta pilbara Watson, 1969	N	U	0.939	0.985	199	100	0	2.5	Y	5	1	100	0	2.5	Y	5	1
Nososticta solida (Hagen, 1860)	E	C	0.728	0.888	599998	34	32	1		1	5	49	38	1.5		1	5
Nososticta solitaria (Tillyard, 1906)	E	C	0.783	0.94	149277	18	39	1.5		1	5	27	45	1.5		1	5
Nososticta taracumbi Watson & Theischinger, 1984	N	U	0.937	0.982	1392	98	31	1.5	Y	4	1	98	2	3	Y	2	2

Species	Region	Record Group	Model Score		Current Range	RCP85_2055						RCP85_2085					
			TSS	AUC		Loss	Gain	C	S	D	V	Loss	Gain	C	S	D	V
Notoaeschna geminata Theischinger, 1982	E	C	0.84	0.955	51743	20	105	1.5		1	5	41	87	2.5		2	5
Notoaeschna sagittata (Martin, 1901)	S	C	0.874	1.011	85029	54	34	1.5		1	5	75	25	2	Y	4	1
Notolibellula bicolor Theischinger & Watson 1977	N	U	0.951	0.997	10868	88	182	2		1	5	94	208	2		3	3
Oristicta filicicola Tillyard, 1913	NE	C	0.944	0.985	12059	35	52	0.5		1	0	61	41	1.5		1	5
Orthetrum boumiera Watson & Arthington, 1978	E	U	0.941	0.985	906	84	84	2		1	5	100	81	4		5	3
Orthetrum caledonicum (Brauer, 1865)	C	C	0.687	0.745	6065482	1	1	1		0	5	3	1	1.5		0	5
Orthetrum migratum Lieftinck, 1951	C	C	0.711	0.926	616573	35	67	1.5		1	5	70	51	2		2	5
Orthetrum sabina (Drury, 1770)	C	C	0.745	0.948	204114	10	157	1		1	5	25	61	2.5		1	5
Orthetrum serapia Watson, 1984	NE	C	0.882	0.974	44700	35	89	1.5		1	5	69	97	3		1	5
Orthetrum villosovittatum (Brauer, 1865)	C	C	0.767	0.899	465265	39	26	1		1	5	55	24	1.5		1	5
Pantala flavescens (Fabricius, 1798)	C	C	0.677	0.859	2833165	6	38	1.5		1	5	7	64	2		1	5
Parasynthemis regina (Selys, 1874)	E	C	0.746	0.93	539172	38	29	1		1	5	53	33	2		1	5
Pentathemis membranulata Karsch, 1890	N	C	0.894	0.982	90251	14	331	1.5		1	5	34	202	3		1	5
Petalura gigantea Leach, 1815	S	C	0.891	0.971	22059	80	79	1.5		3	3	97	45	2.5		5	3
Petalura hesperia Watson, 1958	W	U	0.952	0.994	9622	26	18	2.5		1	5	44	16	3		1	5
Petalura ingentissima Tillyard, 1908	NE	C	0.913	0.986	11616	18	102	1		2	5	56	81	1.5		2	5
Petalura litorea Theischinger, 1999	E	U	0.952	0.995	6620	39	62	2		1	5	60	66	4		2	5
Podopteryx selysi (Förster, 1899)	NE	U	0.952	0.996	7570	72	39	1		1	5	96	19	2	Y	1	2
Potamarcha congener (Rambur, 1842)	N	C	0.797	0.938	312330	9	250	1.5		1	5	13	248	2		1	5
Procordulia affinis (Selys, 1871)	W	C	0.891	0.978	882546	64	1	0.5	Y	2	6	69	1	1.5	Y	0	2
Procordulia jacksoniensis (Rambur, 1842)	S	C	0.721	0.937	209815	54	4	0		0	0	68	3	0.5	Y	0	6
Pseudagrion aureofrons Tillyard, 1906	C	C	0.726	0.908	1288735	30	121	1		1	5	43	127	1.5		1	5
Pseudagrion cingillum (Brauer, 1869)	N	C	0.953	0.989	212429	50	466	1.5		1	5	74	323	3		2	5
Pseudagrion ignifer Tillyard, 1906	E	C	0.773	0.95	252537	28	48	1		1	5	42	53	1.5		1	5
Pseudagrion jedda Watson & Theischinger, 1991	N	C	0.817	0.964	219876	39	132	2		1	5	80	46	2		4	3
Pseudagrion lucifer Theischinger, 1997	N	C	0.832	0.954	174071	16	141	2		1	5	43	38	2		1	5
Pseudagrion microcephalum (Rambur, 1842)	C	C	0.722	0.897	949461	14	136	1		1	5	33	157	1.5		1	5
Pseudocordulia circularis Tillyard, 1909	NE	U	0.936	0.982	710	99	61	2	Y	3	1	99	62	2	Y	2	2
Pseudocordulia elliptica Tillyard, 1913	NE	U	0.952	0.995	7631	47	34	1		1	5	70	8	2	Y	1	2
Rhadinosticta banksi (Tillyard, 1913)	N	C	0.953	0.995	216370	42	227	1		1	5	68	90	1.5		2	5
Rhadinosticta simplex (Martin, 1901)	E	C	0.656	0.89	675694	40	32	1		1	5	53	36	2		1	5

Species	Region	Record Group	Model Score		Current Range	RCP85_2055						RCP85_2085					
			TSS	AUC		Loss	Gain	C	S	D	V	Loss	Gain	C	S	D	V
Rhodothemis lieftincki Fraser, 1954	C	C	0.703	0.901	355893	25	146	1		1	5	32	93	1.5		1	5
Rhyothemis braganza Karsch, 1890	N	C	0.737	0.94	303520	18	60	1.5		1	5	35	32	1.5		1	5
Rhyothemis graphiptera (Rambur, 1842)	C	C	0.697	0.881	737526	10	88	1		1	5	10	58	1.5		1	5
Rhyothemis phyllis (Sulzer, 1776)	NE	C	0.798	0.956	149396	9	79	1		1	5	20	123	2.5		1	5
Rhyothemis princeps Kirby, 1894	NE	C	0.873	0.968	22721	32	58	1		1	5	65	25	2.5		1	5
Rhyothemis resplendens Selys, 1878	NE	C	0.935	0.98	11576	35	32	1.5		0	5	52	32	3.5		0	5
Spinaeschna tripunctata (Martin, 1901)	S	C	0.814	0.964	76305	31	72	1		1	5	52	64	2		1	5
Spinaeschna watsoni Theischinger, 1982	NE	U	0.942	0.985	10299	66	4	1	Y	0	2	95	0	2	Y	2	2
Synlestes selysi Tillyard, 1917	E	C	0.802	0.957	89716	44	84	0.5		1	0	68	89	1.5		5	3
Synlestes tropicus Tillyard, 1917	NE	C	0.926	0.965	4248	77	36	1.5		1	5	97	12	2	Y	0	2
Synlestes weyersii Selys, 1869	S	C	0.755	0.922	261308	28	21	1		2	5	48	23	2		2	5
Synthemiopsis gomphomacromioides Tillyard, 1917	S	U	0.942	0.979	3784	94	17	1.5	Y	1	2	99	8	2.5	Y	1	2
Synthemis eustalacta (Burmeister, 1839)	S	C	0.739	0.927	266770	43	25	0.5		1	0	67	24	1.5	Y	4	1
Synthemis tasmanica Tillyard, 1910	S	C	0.765	0.919	24177	58	20	0.5		1	0	76	8	2	Y	1	2
Teinobasis rufithorax (Selys, 1877)	NE	C	0.95	0.995	3435	41	122	1.5		1	5	65	162	3		1	5
Telephlebia brevicauda Tillyard, 1916	S	C	0.804	0.95	72187	47	50	0.5		1	0	63	46	1		4	3
Telephlebia cyclops Tillyard, 1916	E	C	0.862	0.961	50277	44	44	1.5		1	5	72	41	2.5		2	5
Telephlebia godeffroyi Selys, 1883	E	C	0.819	0.98	110546	50	58	2		1	5	71	55	2		3	3
Telephlebia tillyardi Campion, 1916	NE	U	0.942	0.99	9652	78	39	1		1	5	96	19	2	Y	1	2
Telephlebia tryoni Tillyard, 1917	E	U	0.953	0.99	7294	97	49	2	Y	5	1	100	39	3	Y	5	1
Tetrathemis irregularis cladophila Tillyard, 1908	NE	C	0.937	0.986	9666	67	11	0.5	Y	0	6	91	7	2	Y	1	2
Tholymis tillarga (Fabricius, 1798)	N	C	0.824	0.988	113138	11	348	1.5		1	5	23	172	3		1	5
Tonyosynthemis claviculata (Tillyard, 1909)	NE	U	0.932	0.977	6486	59	18	1		0	5	81	2	2	Y	0	2
Tonyosynthemis ofarrelli (Theischinger & Watson, 1986)	E	U	0.934	0.991	16394	60	236	2.5		1	5	96	207	3		2	5
Tramea eurybia Selys, 1878	E	U	0.949	0.994	1396	81	181	3		5	3	83	233	4		5	3
Tramea loewii Kaup, 1866	C	C	0.762	0.893	1394206	3	153	1		1	5	3	197	1.5		1	5
Tramea stenoloba (Watson, 1962)	C	C	0.751	0.953	993288	52	62	1.5		3	3	62	70	2		2	5
Urothemis aliena Selys, 1878	NE	U	0.948	0.982	31745	41	52	1.5		3	3	58	62	3		1	5
Xanthagrion erythroneurum (Selys, 1876)	C	C	0.722	0.822	3840167	29	12	1		0	5	29	18	1.5		1	5
Zephyrogomphus lateralis (Selys, 1873)	W	C	0.954	0.992	18110	45	2	2	Y	0	2	69	1	2	Y	0	2
Zyxomma elgneri Ris, 1913	C	C	0.711	0.921	1184788	37	78	1		1	5	40	70	1.5		1	5

Species	Region	Record Group	Model Score		Current Range	RCP85_2055						RCP85_2085					
			TSS	AUC		Loss	Gain	C	S	D	V	Loss	Gain	C	S	D	V
Zyxomma petiolatum Rambur, 1842	NE	U	0.95	0.989	11191	22	61	1.5		1	5	45	40	3.5		1	5
