# Supplementary material for: Continental-Scale Assessment of Risk to the Australian Odonata from Climate Change
Source: PLoS One. 2014 Feb 13;9(2):e88958. doi: 10.1371/journal.pone.0088958 (PMC3923880; doi:10.1371/journal.pone.0088958)
Supplement: File S1 — Predictor variables and model validation summary. This file contains Table A and Figure S1. Table A, Environmental factors used to predict species distributions and their mean predictive importance (r2). Figure S1, The ensemble model score for each species (n = 270) based on either the area under the receiver operating characteristic curve (AUC), or the True Skill Statistic (TSS). (PDF) [file pone.0088958.s003.pdf]

FILE S1 Predictor variables and model validation summary

Table A Environmental factors used to predict species distributions and their mean predictive importance (r^2). Variables were assigned to species on the basis of broad geographic regions and number of locality records (common on the left side, uncommon on the right).

Variable	Contin-ental	North		North East		East		South		West	
Mean Annual Temperature	0.53	0.41		0.31		0.41		0.45		0.36	0.38
Temperature Seasonality	0.38	0.38	0.35	0.33	0.38	0.40	0.50	0.35	0.39	0.44	
Min. Temp. Coldest Month	0.38	0.33	0.36	0.31	0.42	0.33	0.50	0.28	0.50	0.43	0.33
Precipitation Seasonality	0.32	0.39	0.45	0.47	0.53	0.35	0.52	0.35	0.49	0.39	0.30
Precipitation of Wettest Qtr.	0.41	0.61	0.56	0.66	0.83					0.76	0.54
Precipitation of Driest Qtr.						0.41	0.41	0.44	0.59		
Mean Annual Flow	0.37	0.41	0.50	0.47	0.37	0.48	0.50	0.40	0.30	0.46	0.57
Valley Confinement	0.45	0.43		0.40		0.39		0.46		0.39	

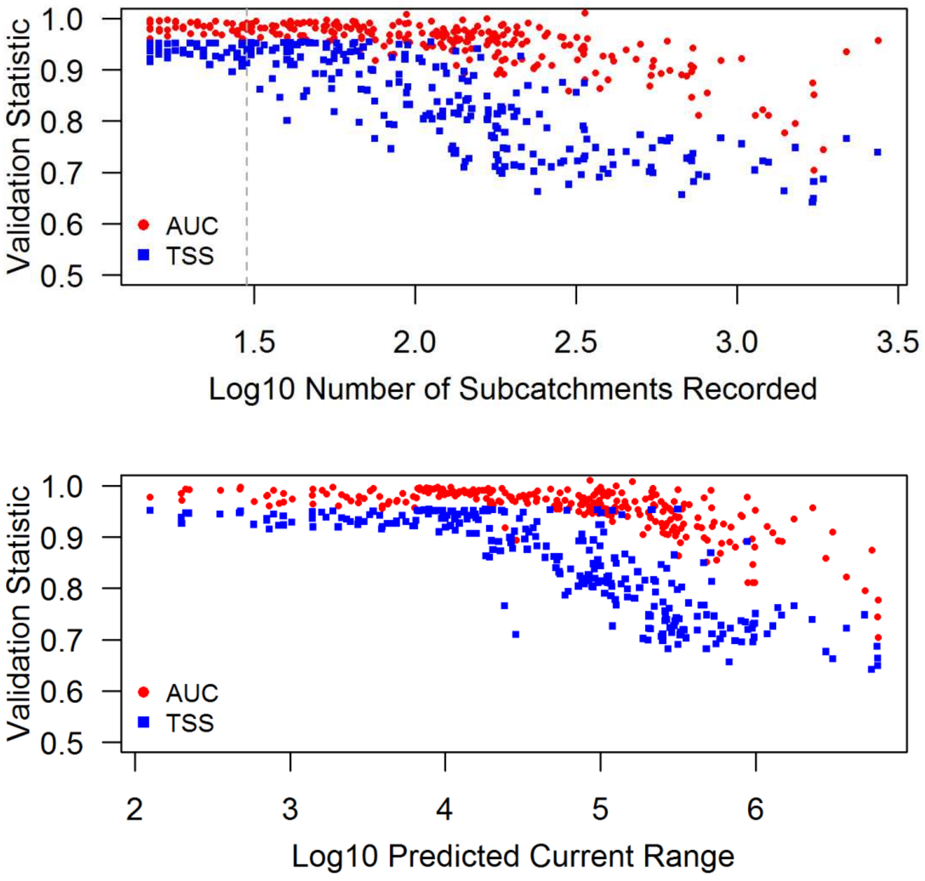

Figure S1dealt with The ensemble model score for each species (n=270) based on either the area under the receiver operating characteristic curve (AUC), or the True Skill Statistic (TSS). The dashed line indicates species with occurrences in 30 subcatchments below which species were modeled as “Uncommon”.
