# Supplementary material for: Continental-Scale Assessment of Risk to the Australian Odonata from Climate Change
Source: PLoS One. 2014 Feb 13;9(2):e88958. doi: 10.1371/journal.pone.0088958 (PMC3923880; doi:10.1371/journal.pone.0088958)
Supplement: File S2 — Demonstration of species vulnerability assessment. This document contains Figure S2–S10 and Table S3–S7. Figure S2, Distribution of occurrence records for Notoaeschna sagittata (green, n = 336), Tetrathemis irregularis cladophila (red, n = 57) and Austrosticta frater (blue, n = 24). Figure S3, Predicted habitat suitability for Notoaeschna sagittata under current and future climatic conditions. Figure S4, Predicted habitat suitability for Tetrathemis irregularis cladophila under current and future climatic conditions. Figure S5, Predicted habitat suitability for Austrosticta frater under current and future climatic conditions. Figure S6, Density plot showing the frequency of habitat suitability scores within the current and future range of Tetrathemis irregularis cladophila. Figure S7, Distance (km) from existing records of a species to all other sites. Figure S8, The probability of dispersal according to distance from occurrence records. Figure S9, Sensitivity weighting for RCP8.5 2085 plotted against dispersal threshold. Figure S10, Categories of vulnerability to climate change effects for species based on three components; exposure, sensitivity and dispersal pressure. Table S3, Predicted change in suitable habitat for two emissions scenarios for 2055 and 2085. Table S4, Summary of scoring system for environmental exposure. Table S5, Environmental exposure scores for Notoaeschna sagittata, Tetrathemis irregularis cladophila and Austrosticta frater. Table S6, Average habitat shifts (km) under climate change scenarios. Table S7, Scoring for the dispersal component of the vulnerability assessment. (PDF) [file pone.0088958.s004.pdf]

File S2. Demonstration of Species Vulnerability Assessment

This supplement provides a detailed description of the information used to assess vulnerability for three of the dragonfly species modelled. We chose species that displayed differing responses to climate change and resulted in different threat categories in the RCP8.5 2085 scenario. These species are: *Notoaeschna sagittata* classified as Highly Vulnerable in Category 1, *Tetrathemis irregularis cladophila* (Vulnerable, Category 2), and *Austrosticta frater* (possible persistence, Category 3).

First, Figure S2 shows the distribution of available records for model building after the dataset had been reviewed and outliers removed. An ensemble of modelling algorithms was then used to predict habitat suitability under current and future climate scenarios, and shows *N. sagittata* is likely to lose suitable habitat in the north of its range and could occupy new habitats in Tasmania in 2085 (Fig. S3). The decline in the current suitable habitat of *T. irregularis cladophila* is proportionally much greater and this species is not predicted to shift to higher latitudes (Fig. S4). Despite some loss of suitability in the current range, the extent of suitable habitat for *Austrosticta frater* is predicted to increase in all scenarios (Fig. S5).

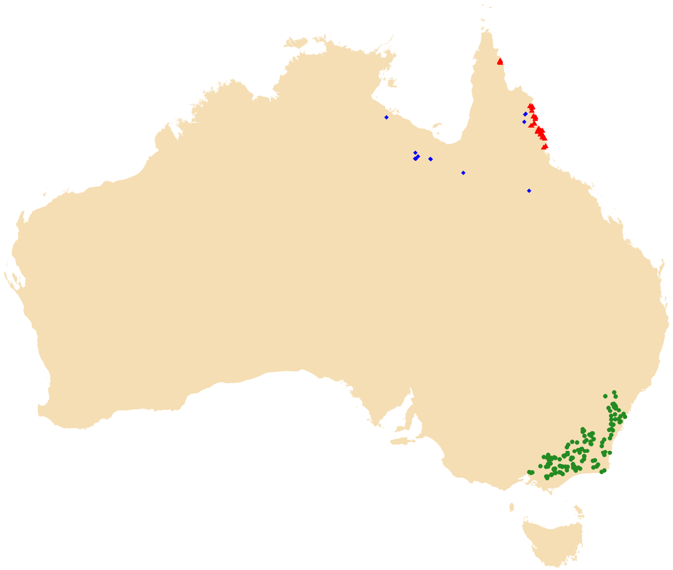

Figure S2 Distribution of occurrence records for *Notoaeschna sagittata* (green, *n*=336), *Tetrathemis irregularis cladophila* (red, *n*=57) and *Austrosticta frater* (blue, *n*=24).

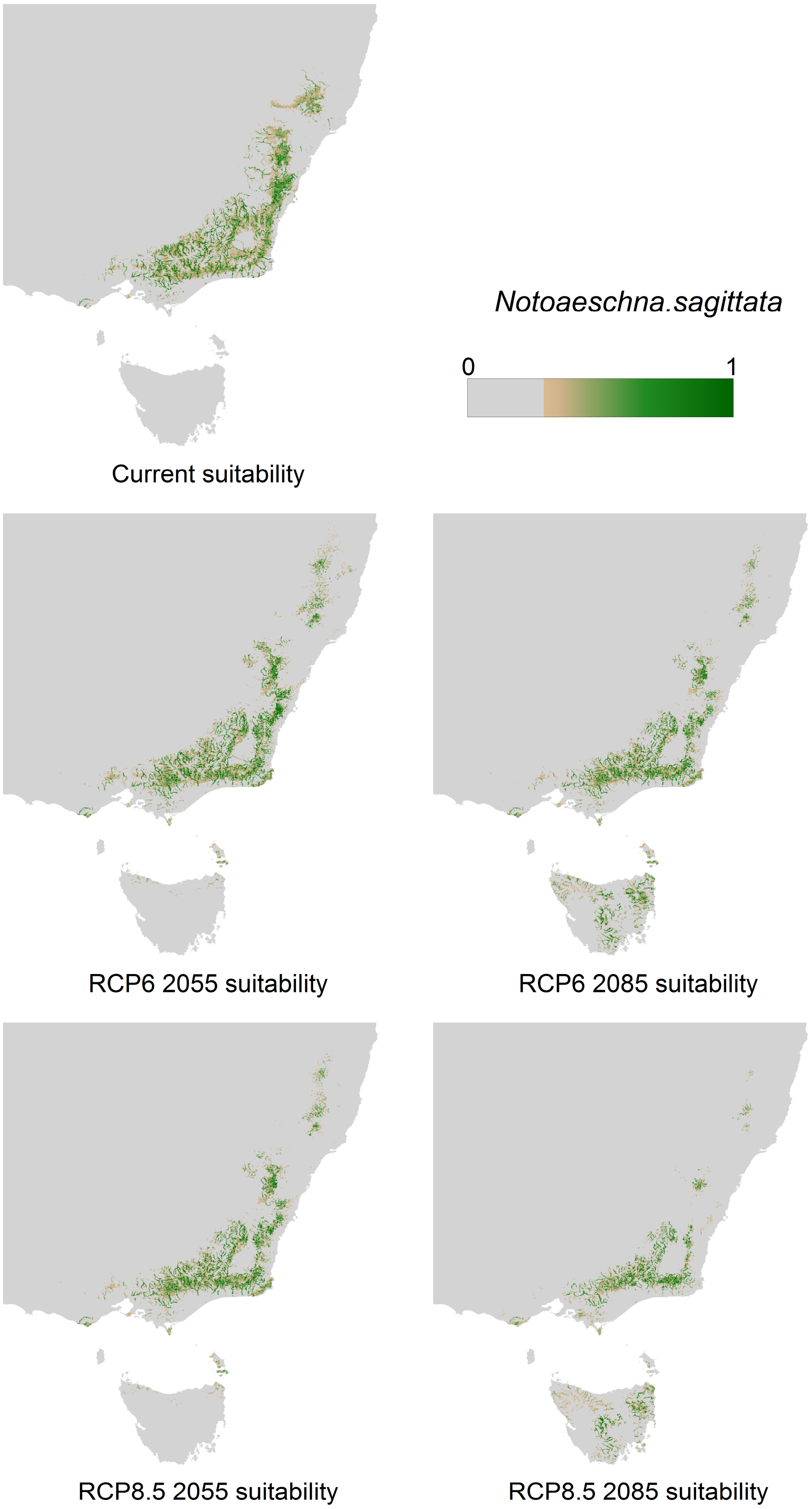

Figure S3 Predicted habitat suitability for *Notoaeschna sagittata* under current and future climatic conditions.

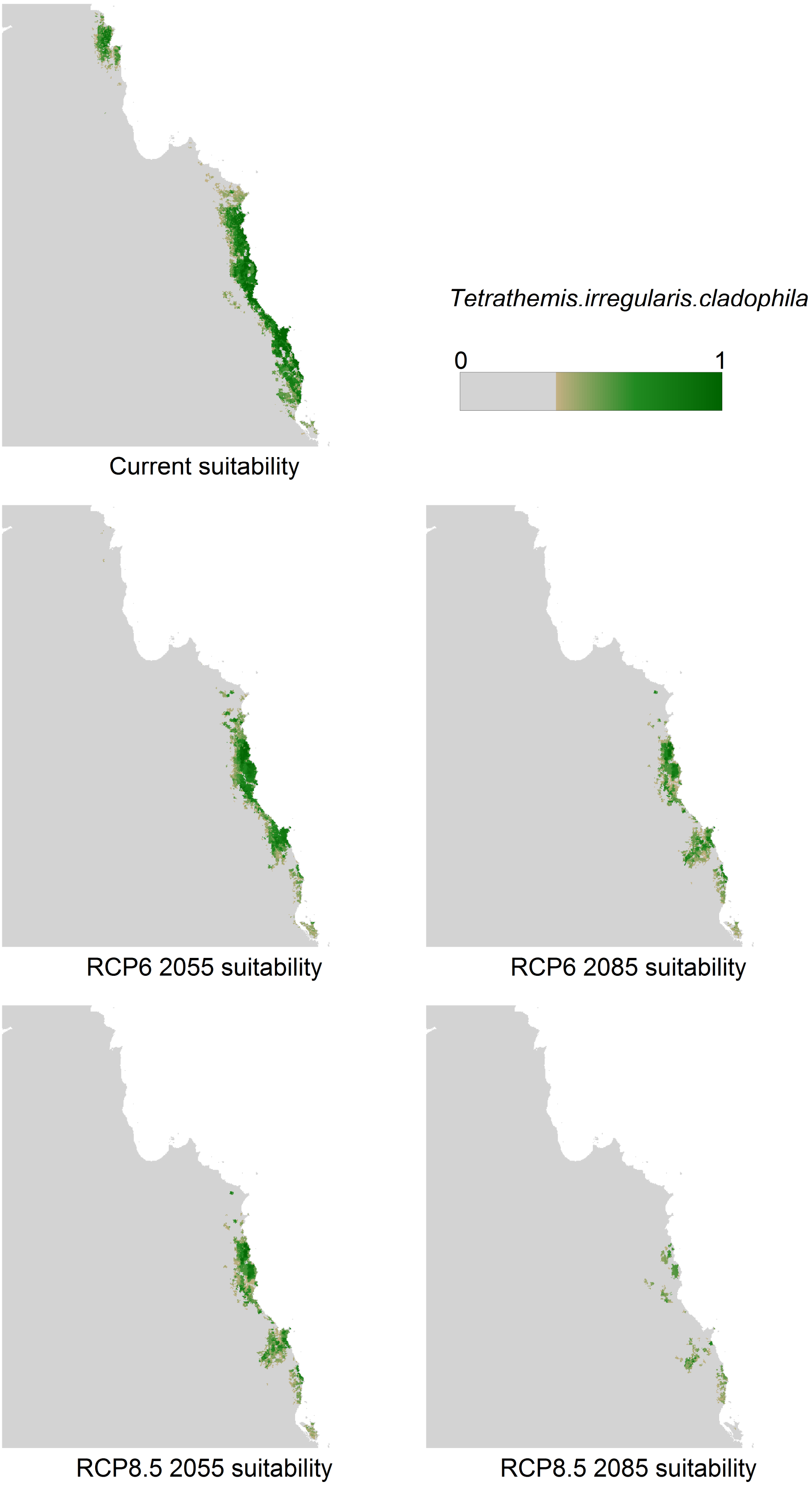

Figure S4 Predicted habitat suitability for *Tetrathemis irregularis cladophila* under current and future climatic conditions.

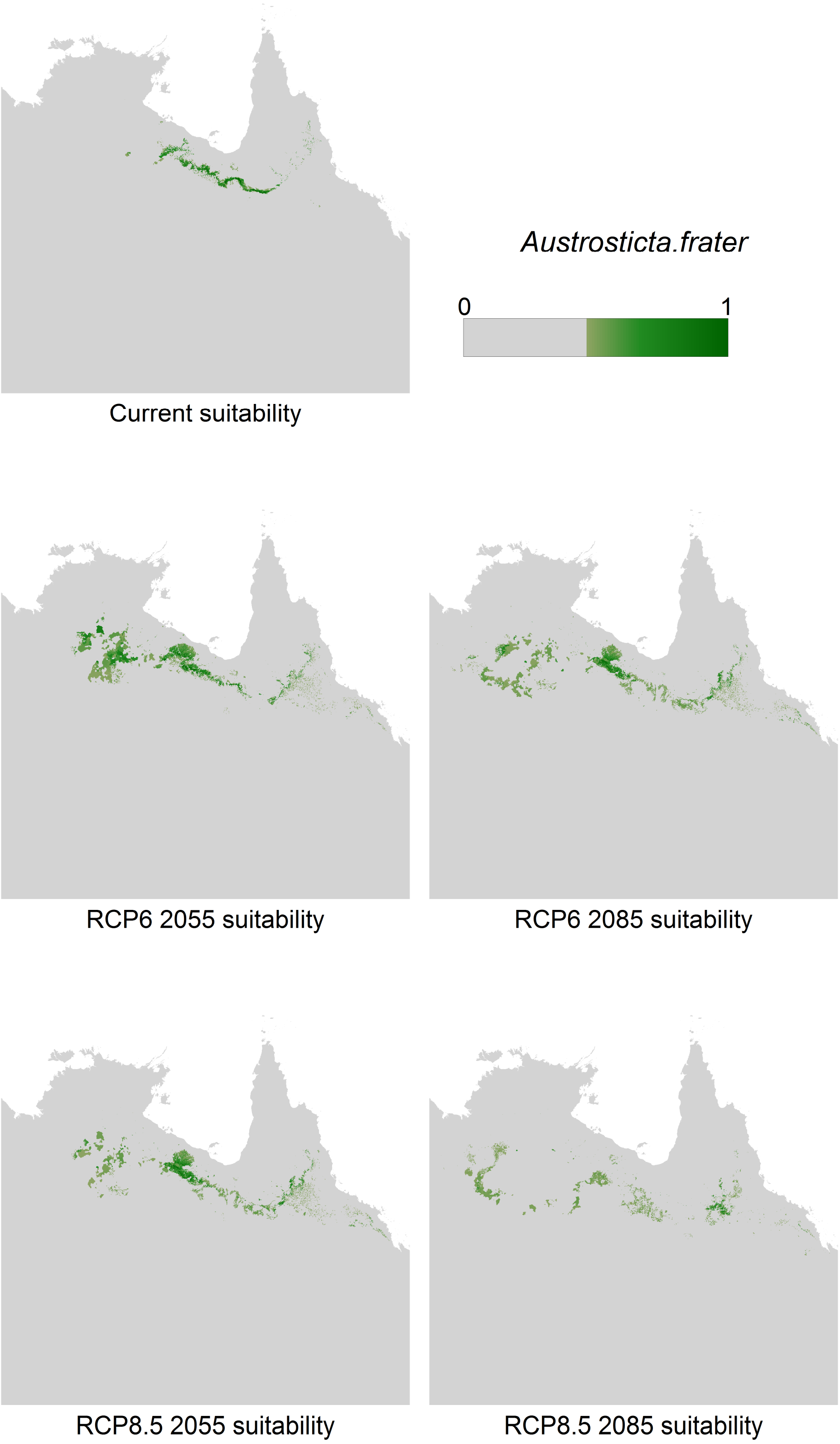

Figure S5 Predicted habitat suitability for *Austrosticta frater* under current and future climatic conditions.

Table S3 Predicted change in suitable habitat for two emissions scenarios for 2055 and 2085. Values are the percentage loss, percentage gain, overall change and sensitivity weighting.

Scenario		*Notoaeschna sagittata*	*T. irregularis cladophila*	*Austrosticta frater*
RCP6_2055	% Loss	39	50	56
	% Gain	24	7	286
	% Change	-15	-43	230
	Sensitivity	0.1598	0.9111	-0.4439
RCP85_2055	% Loss	51	64	61
	% Gain	20	11	227
	% Change	-31	-53	166
	Sensitivity	0.3569	1.8425	-0.5664
RCP85_2055	% Loss	54	67	64
	% Gain	34	11	284
	% Change	-20	-56	220
	Sensitivity	0.4725	1.6474	-0.5058
RCP85_2085	% Loss	75	91	98
	% Gain	25	7	186
	% Change	-50	-84	88
	Sensitivity	1.3499	8.2038	-0.1267

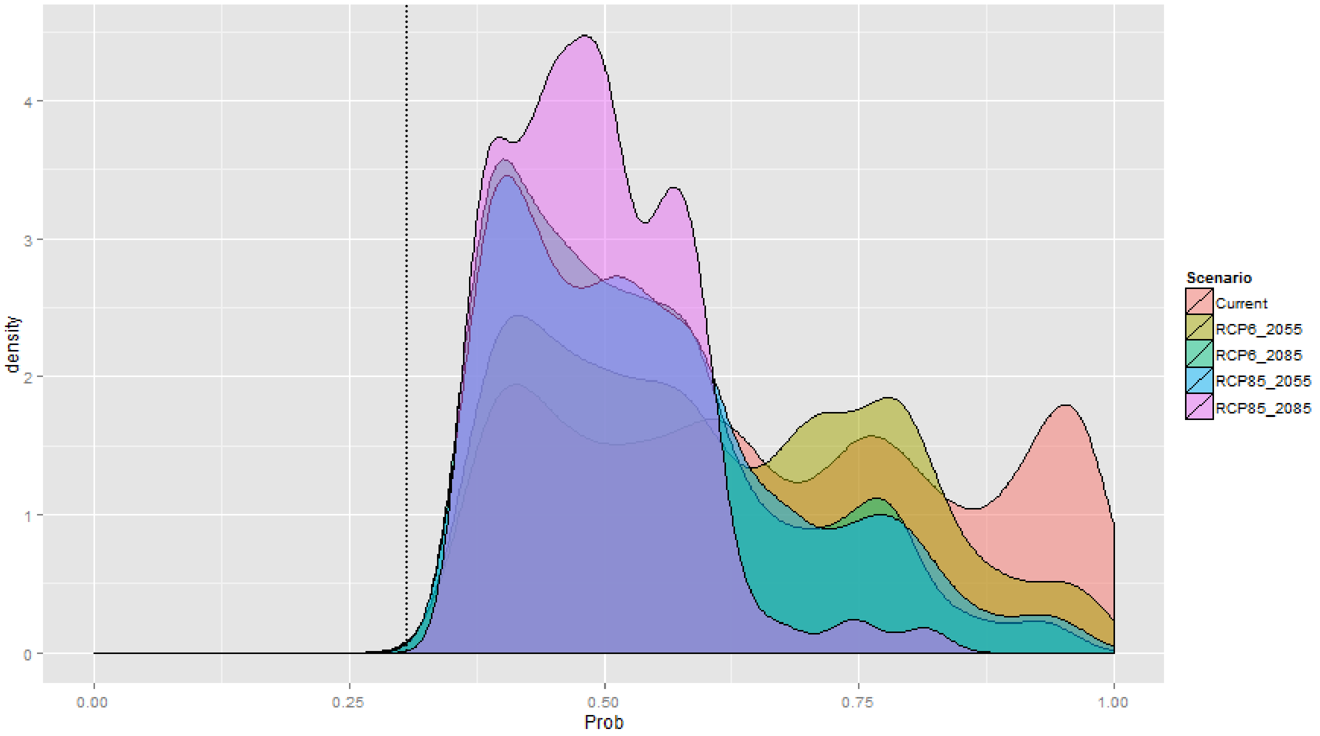

Figure S6 Density plot showing the frequency of habitat suitability scores within the current and future range of *Tetrathemis irregularis cladophila.* Note density is proportional and does not reflect habitat extent. Current habitat suitability includes more high suitability sites than future scenarios and under scenario RCP8.5 2085 the species' remaining habitat is dominated by lower suitability sites. The species is not considered to occur in habitat with suitability values below 0.3 (dashed line).

The vulnerability assessment includes three components: sensitivity (the extent to which a suitable habitat is lost), exposure (the extent to which a species' currently occupied physical environment will change), and dispersal pressure (the reliance on dispersal to avoid further negative impacts) (Fig.1 main document).

SENSITIVITY

Changes to species distribution are summarized in Table S3 and include the sensitivity weights which are the ratio between the change in habitat suitability (sum of habitat suitability over all streams in the future, subtracted from the sum of suitability for streams under current climate), and the current total suitability. *A. frater* loses over 50% of its current suitable habitat but the expansion of new habitats even under harsh climate scenarios means its sensitivity weights are low. Declines in *T. irregularis cladophila* are more severe than for *N. sagittata*, and so its sensitivity weights are higher. For *T. irregularis cladophila*, its extent declines slightly more in RCP8.5 2055 than RCP6 2085 and yet the sensitivity weight is lower. This is because the weighting is based on summed suitability, not the overall change in extent, and in this case although habitat may qualify as sufficiently suitable to support the species, its range overall contains fewer areas with high suitability (Fig. S6). Species with negative sensitivity values are likely to expand their range or have higher overall suitability in the future, whereas higher values occur when the species' habitat either contracts in area, or becomes less suitable. We considered species with a sensitivity score above one to be highly vulnerable.

EXPOSURE

The exposure component of vulnerability was based on the degree to which the physical environment is predicted to change within the current extent of suitable habitat. An average shift of two standard deviations would be equivalent to 97.5% of habitat changing beyond the species current environmental limits. Exposure was based on four climatic factors, one hydrological, and sea level rise (Table S4). Species were considered vulnerable to climatic exposure if the change in one environmental factor was greater than two SDs, or greater than one SD for multiple factors. Sea level rise was considered important if more than 10% of current habitat was below 2m above sea level. In the case of the three example species, none were significantly at risk due to changes in temperature seasonality, mean annual precipitation or sea level rise, but all three were exposed to other environmental factors (Table S5).

Table S4 Summary of scoring system for environmental exposure. A species that scored 1 or more was considered vulnerable for that component.

**Change in Environment =**	**1SD**	**2SD+**
Mean Temperature	0.5	1
Temperature Seasonality	0.5	1
Mean Precipitation	0.5	1
Precipitation Seasonality	0.5	1
Man Annual Flow	0.5	1
**Sea Level Rise**	**<10%**	**>10%**
Current range below 2m	0	0.5

Table S5 Environmental exposure scores for *Notoaeschna sagittata, Tetrathemis irregularis cladophila* and *Austrosticta frater*. Values represent the number of standard deviations future environment will shift from the current habitat average across the species current suitable habitat area. For sea level rise, values are the percentage of current habitat affected by a 1 m rise in sea level.

Species	Factor	RCP6 2055	RCP6 2085	RCP8.5 2055	RCP8.5 2085
N. sagittata	Mean annual temperature	0.67	1.12	1.04	1.82
	Temperature Seasonality	0.32	0.47	0.44	0.71
	Mean annual precipitation	0.05	0.15	0.13	0.28
	Precipitation Seasonality	0.69	1.00	0.95	1.62
	Mean Annual Flow	2.12	4.91	4.22	6.43
	Sea Level Rise	0.36	0.36	0.36	0.36
T. irregularis cladophila	Mean annual temperature	1.17	1.95	1.81	3.19
	Temperature Seasonality	-0.01	0.00	0.00	0.04
	Mean annual precipitation	-0.16	-0.11	-0.12	-0.06
	Precipitation Seasonality	0.48	0.77	0.72	1.24
	Mean Annual Flow	0.51	0.61	0.59	1.02
	Sea Level Rise	6.98	6.98	6.98	6.98
A. frater	Mean annual temperature	1.58	2.57	2.40	4.14
	Temperature Seasonality	0.28	0.23	0.24	0.16
	Mean annual precipitation	-0.50	-0.55	-0.54	-0.65
	Precipitation Seasonality	-0.22	0.75	0.60	2.26
	Mean Annual Flow	1.67	3.30	2.81	8.81
	Sea Level Rise	0.06	0.06	0.06	0.06

DISPERSAL

The third component of the vulnerability assessment is the pressure on a particular species to disperse rapidly in order to occupy suitable habitat in the future. Predicted habitat suitability (Table S3) was modelled under the assumption species would be able to disperse at an average rate of 15 km year^{-1}. Faster expansion consistent with climate change has already been observed in some dragonfly species (e.g. Flenner & Sahlén, 2008). Nonetheless, predicting the success of range shifts over such large distances is highly uncertain (Astorga *et al.*, 2011), and species are potentially at greater risk if their predicted future persistence relies on the assumption of rapid dispersal (Crossman *et al.*, 2011). We therefore split the assessment of dispersal into two parts: distance of habitat shifts, and the dependence of the sensitivity weighting to dispersal thresholds.

The significance of range shifts was assessed using a Wilcoxon rank sum test to determine whether future suitable habitats were significantly further away from point records of that species than the predicted distribution of current suitable habitats. Distances from records were calculated as least cost paths to account for the lower probability that species would cross open seas (Fig. S7). Species scored 0 if suitable habitat shifts were not significant, 1 if the difference was significant (p=0.05 ~2 SDs), and 2 if the difference was over three SDs indicating decreasing habitat overlap. In the case of the three example species, the shift south by *N. sagittata* was significant, but did not cover a significant distance (Table S6). By 2085, new suitable habitats in Tasmania are much further away. The distribution of *T. irregularis cladophila* shrinks but does not show a significant shift. The current range of *A. frater* was predicted to be quite dispersed, and as such although range expansion by 2055 was projected to be extensive, it was only above three SDs in 2085.

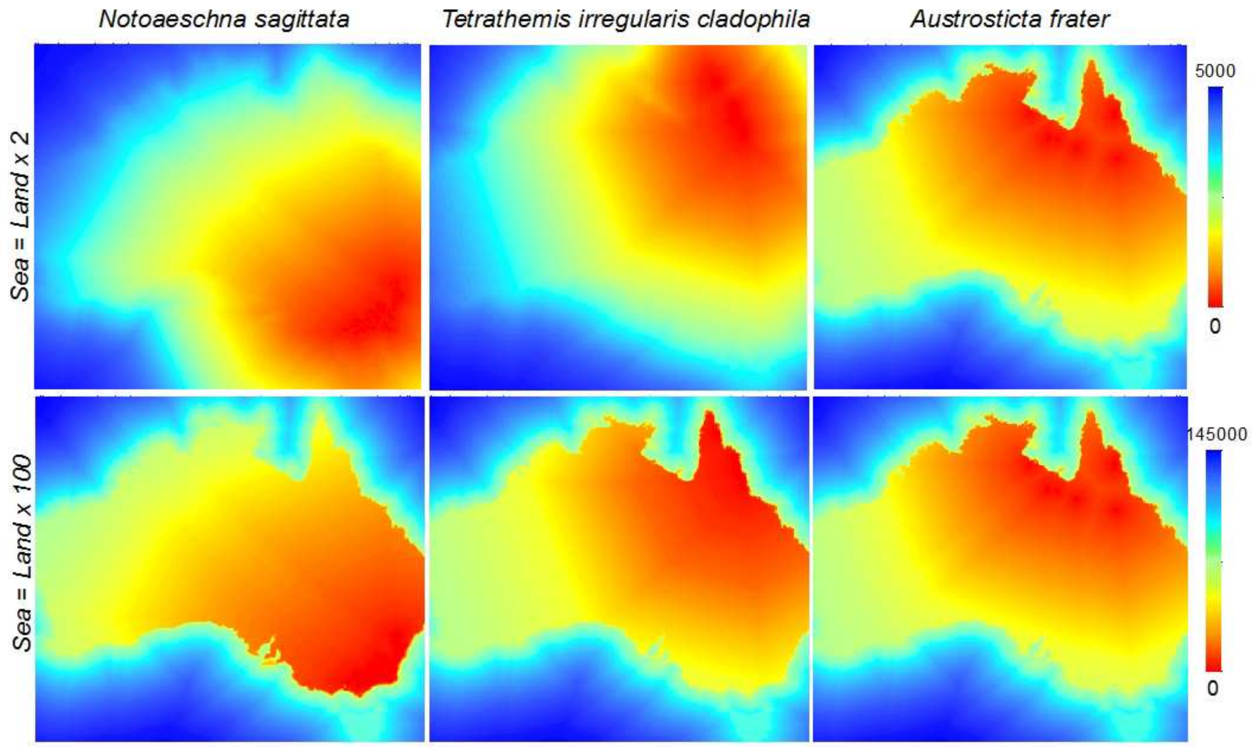

Figure S7 Distance (km) from existing records of a species to all other sites. In the top row, the costs of crossing open water are double that of land used for standard modelling. In the second row, the costs are 100 times greater, making the Bass Strait a dispersal barrier for mainland species shifting to Tasmania.

Table S6 Average habitat shifts (km) under climate change scenarios. Distances marked with a * were significantly further than the current habitat distribution, and ** if the change was over three standard deviations further.

Species	Current	RCP6 2055	RCP6 2085	RCP8.5 2055	RCP8.5 2085
Notoaeschna sagittata	34	60 *	170 *	53 *	222 **
Tetrathemis irregularis cladophila	16	14	14	14	16
Austrosticta frater	92	249 *	293 **	210 *	363 **

To account for the uncertainty in a species ability to disperse, we considered the effect on a species overall habitat suitability, using the sensitivity weighting, when dispersal rates were gradually reduced. Suitable habitat was constrained by dispersal using the distances from observed records (Fig. S7) and a dispersal kernel (see Fig. 2, main document). The dispersal kernel is a four-parameter logistic curve that converts all distances to a value between 0 and 1, interpreted as the estimated probability a species could disperse to that site. The dispersal kernel can be modified depending on a threshold distance, and the rate of decay (Fig. S8).

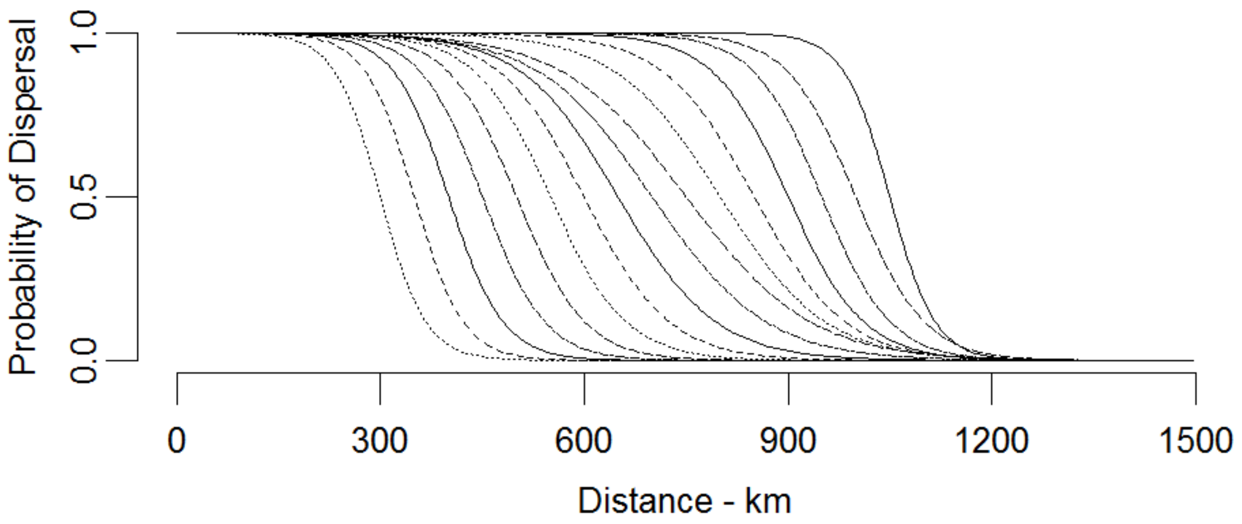

Figure S8 The probability of dispersal according to distance from occurrence records. By iteratively reducing the threshold or rate of decay, the dispersal constraint increasingly restricts the suitable habitat within dispersal (note not all levels are shown).

As the overall limit of dispersal is reduced, habitat suitability is reduced at distant locations from species current records, and as a consequence, the suitability weighting increases. Reducing the rate of dispersal by increments of 0.5 km year^{-1} provides 30 dispersal thresholds, which we divided into three groups (10.5-15 km year^{-1} = High dispersal, 5.5-10 km year^{-1} = Medium dispersal, and 0.5-5 km year^{-1} = Low dispersal). The rate at which sensitivity weight increased was determined from the slope of a linear model between the threshold distance and the sensitivity weighting (note distances were different for 2055 and 2085) (Fig. S9). The effect of a reduction in dispersal capacity on a species suitable habitat is relative to its overall sensitivity weighting for each emission scenario, but was considered significant if the slope was less than -1. A species was given a score of 3, 2 or 1 if the slope was less than one for high, medium or low thresholds respectively, and zero if it was not.

In the case of the three example species, the extent and suitability of habitat for *T. irregularis cladophila* would not be at greater risk if its dispersal ability was constrained. A small reduction in the dispersal capacity may not greatly affect *N. sagittata* either, but if dispersal rates were reduced below ~11 km year^{-1}, the overall quantity of suitable habitat available quickly decreases, observed here as a rapid increase in sensitivity weighting (Fig. S9a, green line, score =2). A rapid increase in the sensitivity weighting only occurred for *A. frater* if the dispersal rate was heavily reduced (Fig. S9c, blue line, score=1) and so there is only a small risk it will not be able to occupy the majority of suitable habitats available.

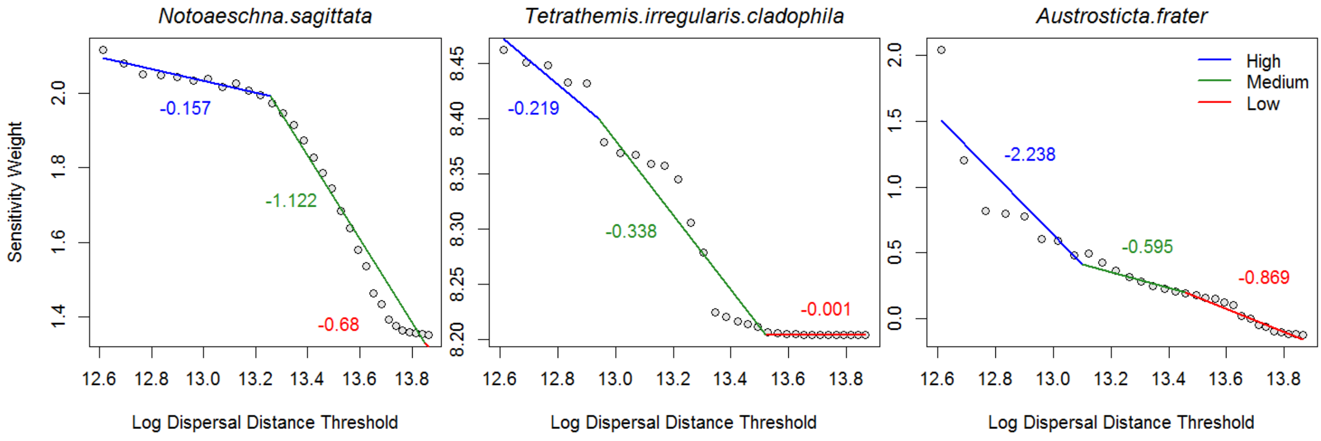

Figure S9 Sensitivity weighting for RCP8.5 2085 plotted against dispersal threshold (distance in metres log transformed). Values are the slope of a linear regressions fitted to scores for high (blue), medium (green) and low (red) rates of dispersal (*n*=10).

The scores allocated for the distance of habitat shifts (0-2) and reliance on dispersal (0-3) provide a maximum of 5 points and species that scored three or more in a particular climate scenario were considered as vulnerable for this component of the assessment (Table S7). Thus, all species whose sensitivity would increase rapidly from minor reductions in the dispersal threshold are considered vulnerable. For example in 2085 RCP8.5, *N. sagittata* was considered vulnerable based on dispersal because it is predicted to both shift a significant distance from its current distribution (Table S6), and the majority of its suitable habitat will be at risk if it cannot maintain at least moderately high rates of dispersal (Fig. S9, Table S7).

Table S7 Scoring for the dispersal component of the vulnerability assessment. Species that score three or more in any climate scenario (shaded) are considered at risk.

		Sensitivity to Dispersal Threshold			
		0	1	2	3
Score for Habitat Shift	0	0	1	2	3
	1	1	2	3	4
	2	2	3	4	5

Thus we have three components that address vulnerability under climate change. Species at risk across all components are most vulnerable (Category 1) but species under less pressure to disperse are still considered vulnerable (Category 2) (Fig. S10). If a species is exposed to climate change and alternative suitable habitats require significant dispersal, they are considered potential persisters (Category 3). Theoretically habitat suitability could decline and shift without high environmental exposure, but this is highly unlikely using modeled predictions (Category 4).

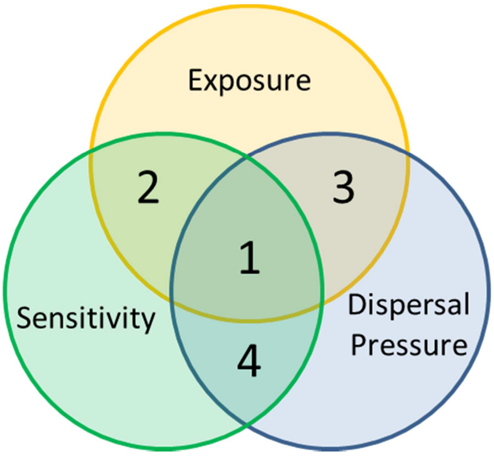

Figure S10 - Categories of vulnerability to climate change effects for species based on three components; exposure, sensitivity and dispersal pressure.

In summary;

- *Notoaeschna sagittata* was exposed in all future climate scenarios, as was *Austrosticta frater* (Table S5). *Tetrathemis irregularis cladophila* was only significantly exposed in the RCP8.5 2085 scenario, although note mean annual temperature was close to two SDs in RCP6 2085, and RCP8.5 2055.
- None of the three species had a high sensitivity weighting under RCP6 2055, but *T.irregularis cladophila* was considered sensitive in all three remaining scenarios. The sensitivity weighting was only high for *N. sagittata* under RCP8.5 2085; *A. frater* did not decline and so was not considered "sensitive" (Table S3).
- For the dispersal component, *N. sagittata* was only significantly at risk under the RCP8.5 2085. The range of *T. irregularis cladophila* is predicted to contract *in-situ* and so it would not rely on high rates of dispersal. *A. frater* is predicted to avoid overall loss of suitable habitat by expanding its range into new areas. Consequently, suitable habitat shifts in all scenarios (Table S6), and in the RCP8.5 2085 scenario there is a risk it will experience significant declines if it fails to sustain at least low rates of dispersal (Fig. S9).
- In most scenarios *N. sagittata* is only considered exposed, but under the RCP8.5 2085 scenario it projected to be at risk for all components and is classed as "Highly Vulnerable" (Category 1, Fig. S10). *T. irregularis cladophila* is not considered threatened at all in RCP6 2055, and only sensitive in RCP6 2085 and RCP8.5 2055. However under RCP8.5 2055 it is both highly sensitive and highly exposed and considered "Vulnerable" (Category 2). Like *N. sagittata*, *A. frater* is only threatened by its exposure for most scenarios, but in 2085 additional distances shifted by its habitat and the implications for dispersal capacity means it is assigned to Category 3 for lower risk species that have potential to persist.

References

Astorga, A., Heino, J., Luoto, M. & Muotka, T. (2011) Freshwater biodiversity at regional extent: determinants of macroinvertebrate taxonomic richness in headwater streams. *Ecography*, **34**, 705-713.

Crossman, N.D., Bryan, B.A. & Summers, D.M. (2011) Identifying priority areas for reducing species vulnerability to climate change. *Diversity and Distributions*, no-no.

Flenner, I.D.A. & Sahlén, G. (2008) Dragonfly community re-organisation in boreal forest lakes: rapid species turnover driven by climate change? *Insect Conservation and Diversity*, **1**, 169-179.
